# Real-world evaluation of rapid and laboratory-free COVID-19 triage for emergency care: external validation and pilot deployment of artificial intelligence driven screening

**DOI:** 10.1016/S2589-7500(21)00272-7

**Published:** 2022-03-09

**Authors:** Andrew A S Soltan, Jenny Yang, Ravi Pattanshetty, Alex Novak, Yang Yang, Omid Rohanian, Sally Beer, Marina A Soltan, David R Thickett, Rory Fairhead, Tingting Zhu, David W Eyre, David A Clifton, CURIAL Translational Collaborative, Adam Watson, Akshay Bhargav, Alex Tough, Alice Rogers, Ayisha Shaikh, Carolina Valensise, Charlotte Lee, Claire Otasowie, David Metcalfe, Ekta Agarwal, Elham Zareh, Evelyn Thangaraj, Florence Pickles, Gabriella Kelly, Gayatri Tadikamalla, George Shaw, Heather Tong, Hettie Davies, Jasdeep Bahra, Jessica Morgan, Joe Wilson, Joseph Cutteridge, Katherine O'Byrne, Luiza Farache Trajano, Madeleine Oliver, Maria Pikoula, Maya Mendoza, Melissa Keevil, Muhammad Faisal, Natasha Dole, Oscar Deal, Rebecca Conway-Jones, Shajeel Sattar, Sneha Kundoor, Sumaiyah Shah, Vani Muthusami

**Affiliations:** a John Radcliffe Hospital, Oxford University Hospitals NHS Foundation Trust, Oxford, UK; b Emergency Medicine Research Oxford, Oxford University Hospitals NHS Foundation Trust, Oxford, UK; c Division of Cardiovascular Medicine, Radcliffe Department of Medicine, University of Oxford, Oxford, UK; d Institute of Biomedical Engineering, Department of Engineering Science, University of Oxford, Oxford, UK; e Big Data Institute, Nuffield Department of Population Health, University of Oxford, Oxford, UK; f Medical School, University of Oxford, Oxford, UK; g The Queen Elizabeth Hospital, University Hospitals Birmingham NHS Foundation Trust, Birmingham, UK; h Institute of Inflammation and Ageing, University of Birmingham, Birmingham, UK; i NIHR Health Protection Research Unit in Healthcare Associated Infections and Antimicrobial Resistance, University of Oxford and Public Health England, Oxford, UK

## Abstract

**Background:**

Uncertainty in patients' COVID-19 status contributes to treatment delays, nosocomial transmission, and operational pressures in hospitals. However, the typical turnaround time for laboratory PCR remains 12–24 h and lateral flow devices (LFDs) have limited sensitivity. Previously, we have shown that artificial intelligence-driven triage (CURIAL-1.0) can provide rapid COVID-19 screening using clinical data routinely available within 1 h of arrival to hospital. Here, we aimed to improve the time from arrival to the emergency department to the availability of a result, do external and prospective validation, and deploy a novel laboratory-free screening tool in a UK emergency department.

**Methods:**

We optimised our previous model, removing less informative predictors to improve generalisability and speed, developing the CURIAL-Lab model with vital signs and readily available blood tests (full blood count [FBC]; urea, creatinine, and electrolytes; liver function tests; and C-reactive protein) and the CURIAL-Rapide model with vital signs and FBC alone. Models were validated externally for emergency admissions to University Hospitals Birmingham, Bedfordshire Hospitals, and Portsmouth Hospitals University National Health Service (NHS) trusts, and prospectively at Oxford University Hospitals, by comparison with PCR testing. Next, we compared model performance directly against LFDs and evaluated a combined pathway that triaged patients who had either a positive CURIAL model result or a positive LFD to a COVID-19-suspected clinical area. Lastly, we deployed CURIAL-Rapide alongside an approved point-of-care FBC analyser to provide laboratory-free COVID-19 screening at the John Radcliffe Hospital (Oxford, UK). Our primary improvement outcome was time-to-result, and our performance measures were sensitivity, specificity, positive and negative predictive values, and area under receiver operating characteristic curve (AUROC).

**Findings:**

72 223 patients met eligibility criteria across the four validating hospital groups, in a total validation period spanning Dec 1, 2019, to March 31, 2021. CURIAL-Lab and CURIAL-Rapide performed consistently across trusts (AUROC range 0·858–0·881, 95% CI 0·838–0·912, for CURIAL-Lab and 0·836–0·854, 0·814–0·889, for CURIAL-Rapide), achieving highest sensitivity at Portsmouth Hospitals (84·1%, Wilson's 95% CI 82·5–85·7, for CURIAL-Lab and 83·5%, 81·8–85·1, for CURIAL-Rapide) at specificities of 71·3% (70·9–71·8) for CURIAL-Lab and 63·6% (63·1–64·1) for CURIAL-Rapide. When combined with LFDs, model predictions improved triage sensitivity from 56·9% (51·7–62·0) for LFDs alone to 85·6% with CURIAL-Lab (81·6–88·9; AUROC 0·925) and 88·2% with CURIAL-Rapide (84·4–91·1; AUROC 0·919), thereby reducing missed COVID-19 cases by 65% with CURIAL-Lab and 72% with CURIAL-Rapide. For the prospective deployment of CURIAL-Rapide, 520 patients were enrolled for point-of-care FBC analysis between Feb 18 and May 10, 2021, of whom 436 received confirmatory PCR testing and ten (2·3%) tested positive. Median time from arrival to a CURIAL-Rapide result was 45 min (IQR 32–64), 16 min (26·3%) sooner than with LFDs (61 min, 37–99; log-rank p<0·0001), and 6 h 52 min (90·2%) sooner than with PCR (7 h 37 min, 6 h 5 min to 15 h 39 min; p<0·0001). Classification performance was high, with sensitivity of 87·5% (95% CI 52·9–97·8), specificity of 85·4% (81·3–88·7), and negative predictive value of 99·7% (98·2–99·9). CURIAL-Rapide correctly excluded infection for 31 (58·5%) of 53 patients who were triaged by a physician to a COVID-19-suspected area but went on to test negative by PCR.

**Interpretation:**

Our findings show the generalisability, performance, and real-world operational benefits of artificial intelligence-driven screening for COVID-19 over standard-of-care in emergency departments. CURIAL-Rapide provided rapid, laboratory-free screening when used with near-patient FBC analysis, and was able to reduce the number of patients who tested negative for COVID-19 but were triaged to COVID-19-suspected areas.

**Funding:**

The Wellcome Trust, University of Oxford Medical and Life Sciences Translational Fund.

**Research in context**

**Evidence before this study**

A recent study estimated that 11·3% of patients in hospital with COVID-19 during the first pandemic wave contracted the virus after admission, recognising triage failure and long PCR turnaround times as contributors. Many works, using both conventional and artificial intelligence approaches, have aimed to improve COVID-19 triage safety. We searched PubMed and Google Scholar from inception up to Sept 30, 2021, for items in English, using the search terms “Covid-19”, “SARS-CoV-2”, “Emergency Department”, “identify” and “triage”, identifying 15 200 items, of which 1870 reported use of artificial intelligence (additional search terms used were “Machine Learning” or “Artificial Intelligence”). Lateral flow testing has been widely adopted, but recent studies showed poor sensitivity for hospital triage (about 62%). Although many artificial intelligence studies reported promising results, two key reviews that, together, screened 40 000 titles, highlighted sector-wide methodological and reporting concerns. Few studies did adequate validation or showed added value in a real-world setting. In 2020, our group reported that an artificial intelligence test (CURIAL-1.0) rapidly identified patients attending the emergency department with COVID-19, using routine blood tests, blood gas, and vital signs collected within 1 h of presentation. The approach was highlighted in a *Lancet Digital Health* editorial, which called for diverse and inclusive evaluation alongside evidence of tangible clinical improvement.

**Added value of this study**

We present a multicentre validation and prospectively deploy an artificial intelligence screening model in a UK emergency department. To our knowledge, our study reports the shortest time from arrival in hospital to an artificial intelligence-driven COVID-19 screening result in a real-world clinical setting, using point-of-care blood testing to achieve results in a median 45 min. High negative predictive values allow the model to be used as a rapid rule-out test, supporting time-critical decision making. An assessment of operational effects showed that the model correctly ruled out infection for 58·5% of patients who were triaged by a clinician or physician to a COVID-19-suspected area but went on to test negative by PCR. Our multicentre validation used cohorts inclusive of all adult emergency admissions to four National Health Service trusts, showing consistent performance across the full length of the COVID-19 pandemic. We found no evidence of consistent biases by gender or ethnic group. Our study also validates a triage pathway that combines an artificial intelligence test with LFDs in the real-world clinical setting, showing that missed COVID-19 cases were reduced from 43 per 100 patients with a positive test when LFDs were used alone to 12 (a 72% reduction). Moreover, this integrated approach offers a framework for low-risk deployment of clinical artificial intelligence, using a pathway that is at least as safe as current practice by design. Together, our findings evidence the generalisability, efficacy, and real-world operational benefits of artificial intelligence-driven screening for COVID-19 in emergency care.

**Implications of all the available evidence**

Whereas competing screening approaches frequently require additional testing, the CURIAL models provide a rapid test-of-exclusion for COVID-19 by use of readily available clinical data. Benefits include the potential to expedite transfers from the emergency department to COVID-19-free clinical areas, thereby reducing operational strain and nosocomial transmission among the inpatient population. The CURIAL models are rapidly scalable, near-universally applicable in the population of intended use, and extensively validated by use of real-world emergency department populations that are easily definable and reproducible. Moreover, the additional cost is negligible, and a high negative predictive value configuration allows targeting of confirmatory testing to subpopulations with higher risk of a positive test. This study contributes to a growing evidence base for responsible use of clinical artificial intelligence tools.

## Introduction

Reducing nosocomial transmission of SARS-CoV-2 is a priority in safeguarding patient and health-care staff safety, particularly as inpatients are at greatest risk of severe illness and death.^1^, ^2^ Although viral testing is mandated for all patients admitted to UK hospitals, long turnaround times and triage failure can cause delays to care, nosocomial transmission, and operational strain in the emergency department.^3^, ^4^, ^5^

The mainstay of testing for SARS-CoV-2 is batch-processed laboratory PCR, which has imperfect sensitivity and requires specialist equipment.^6^, ^7^ Turn-around times have shortened throughout the pandemic, typically to within 12–24 h in hospitals in high-income and middle-income countries, but the interim uncertainty about patients' COVID-19 status might postpone safe transfers from the emergency department to SARS-CoV-2-free clinical areas and thereby contribute to nosocomial transmission.^1^, ^8^ Novel rapid testing solutions have been adopted, including point-of-care PCR, loop mediated isothermal amplification, and antigen testing with lateral flow devices (LFDs), despite limitations in throughput and sensitivity.^9^, ^10^ Where point-of-care PCR is available, its use is typically constrained to time-critical decisions due to supply concerns.^11^, ^12^ Moreover, although LFDs are laboratory-free and highly specific (>99·5%),^13^ multiple reports have shown more limited sensitivity (about 40–70%),^14^, ^15^ leading to the US Food and Drug Administration issuing a class 1 recall of the Innova SARS-CoV-2 rapid antigen test on June 10, 2021.^16^ A recent study showed low sensitivity of LFDs (62%) when used for emergency hospital admissions,^10^ increasing the risk of triage failure.

The subjective assessment of routinely collected blood test results, which are typically available within 1 h of presentation to hospitals in high-income and middle-income countries, is widely used to guide preliminary triage. Although associations such as that between lymphopenia and COVID-19 are well recognised, in isolation these have been shown to be insufficiently sensitive for use as a COVID-19 screening test.^17^ Many studies have characterised complex patterns of viral-induced abnormalities in haematology biochemistry panels,^18^, ^19^ and multivariate clinical scores such as the Corona-score have been developed to support COVID-19 triage in the emergency department.^20^ However, owing to limited specificity of individual predictors, artificial intelligence approaches applied to richer predictor sets are necessary to achieve clinically-acceptable performance.^21^

We have shown that an artificial intelligence screening test (CURIAL-1.0) rapidly identified patients attending the emergency department with COVID-19, using the routine blood test, blood gas, and vital signs collected within 1 h of presentation to the hospital.^5^ Strengths of the approach include the use of readily available and near-universally collected data, thereby being widely applicable, and high negative predictive value (NPV) configuration to offer a rapid rule out. By contrast, alternative approaches to COVID-19 triage use radiological imaging, which is less readily available and involves patient exposure to ionising radiation,^22^ or infrequently requested laboratory predicting markers, such as IL-6.^23^, ^24^ Although many studies have examined diagnostic applications of artificial intelligence for COVID-19, key reviews have highlighted sector-wide methodological and reporting concerns and called for rigorous evaluation within the clinical context of intended use.^25^, ^26^

A 2021 editorial highlighted the promise of CURIAL-1.0 to support patients with COVID-19, discussing the need for further evaluation to show benefits over standard of care in a real-world clinical setting.^27^ Moreover, recent advances in point-of-care testing include the availability of rapid haematology analysers, which offer results within 10 min,^28^ and their role within artificial intelligence-driven COVID-19 screening remains unexplored.

In this study, we aimed to improve the time from arrival to the emergency department to the availability of an artificial intelligence result and evaluate the resultant models. To assess generalisability, we did external and prospective validations across emergency admissions to four UK National Health Service (NHS) trusts, and we compared performance with that of LFDs used in standard care. Lastly, we deployed a model alongside an approved point-of-care full blood count (FBC) analyser (OLO, SightDiagnostics, Tel Aviv, Israel) to provide laboratory-free COVID-19 screening in the John Radcliffe Hospital's Emergency Department (Oxford, UK), assessing time-to-result and diagnostic performance in a real-world clinical setting.

## Methods

### Diagnostic models to identify patients presenting with COVID-19

We updated our previously described model, designed to identify patients presenting to hospital with COVID-19 by use of vital signs, blood gas, and routine laboratory blood tests (CURIAL-1.0),^5^ with additional training data to encompass all COVID-19 cases presenting to Oxford University Hospitals (OUH; Oxfordshire, UK) during the first pandemic wave (to June 30, 2020; appendix pp 3–8). OUH consists of four teaching hospitals serving a population of 600 000 and provides tertiary referral services to the surrounding region. The routine blood tests used were FBC; urea, creatinine, and electrolytes; liver function tests (LFTs); coagulation; and C-reactive protein (CRP), owing to their ubiquity in emergency care pathways and rapid results, typically available within 1 h.

**Figure 1:**
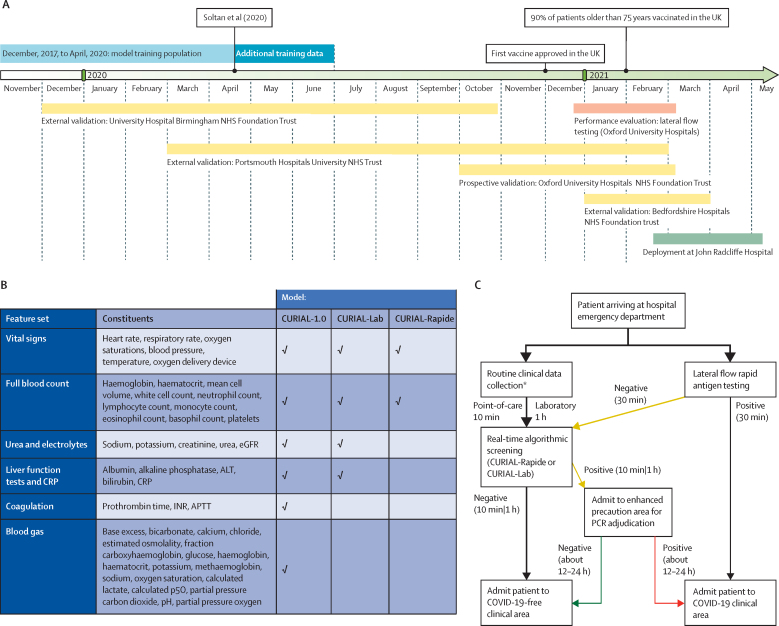
Overview of study design Overview shows the timeline of model development, evaluation, and deployment (A); successive elimination of less informative predictors from CURIAL-1.0 to optimise for generalisability (CURIAL-Lab) and result-time (CURIAL-Rapide; B); and a proposed novel rapid screening pathway for COVID-19 in emergency departments, which combines lateral flow device testing with artificial intelligence screening (C). Routine blood tests and vital signs recordings are done on arrival to the emergency department, either using rapid point-of-care haematology analysers (about 10 min; CURIAL-Rapide) or the existing laboratory pathway (about 1 h; CURIAL-Lab). Real-time algorithmic analysis allows early, high-confidence identification of patients who are negative for safe triage to COVID-19-free clinical areas. Patients with positive CURIAL results are admitted to enhanced precautions (amber) areas, pending confirmatory PCR. Patients testing positive with a lateral flow test are streamed directly to COVID-19 (red) clinical areas. Arrow thickness represents patient flow. ALT=alanine aminotransferase. APTT=activated partial thromboplastin time. CRP=C-reactive protein. eGFR=estimated glomerular filtration rate. INR=international normalised ratio. NHS=National Health Service. p50=pressure at which haemoglobin is 50% bound to oxygen. * CURIAL-Lab used data collected from routine blood tests (full blood count; urea, creatinine, and electrolytes; liver function tests; and C-reactive protein) and vital signs, whereas CURIAL-Rapide used data that could be collected at the patient's bedside (full blood count and vital signs).

Next, we eliminated weakly informative predictors to improve generalisability (figure 1B). CURIAL-Lab, an updated model, uses a focused subset of routine blood tests (FBC; urea, creatinine, and electrolytes; LFTs; and CRP) and vital signs, eliminating the use of coagulation panels and blood gas, which are not universally performed and are less informative.^5^ Separately, we optimised for time-to-result and developed a minimalist model (CURIAL-Rapide) considering only predictors that can be rapidly obtained by the patient bedside (FBC and vital signs). We selected FBC due to the approval of a point-of-care haematology analyser with a time-to-result of 10 min (OLO) and explainability analyses showing that FBC components were most informative (eg, basophil, eosinophil, and neutrophil counts).^5^, ^28^ The timeline of model development, evaluation, and deployment is shown in figure 1A.

NHS Health Research Authority approval was granted for the use of routine clinical and microbiology data from electronic health records (EHRs) for development and validation of artificial intelligence models to detect COVID-19 (CURIAL; IRAS ID 281832). The study protocol for model development and external and prospective validation was approved by the NHS Health Research Authority (IRAS ID 281832) and sponsored by the University of Oxford. The OLO analysers were deployed in an OUH emergency department as service improvement with approvals from the point-of-care testing committee and trust approval for service evaluation and improvement (OUH Ulysses ID 6709).

### Evaluation for the second wave of the UK COVID-19 epidemic at OUH

We evaluated the performance of CURIAL-1.0, CURIAL-Lab, and CURIAL-Rapide by use of an independent prospective set of all patients presenting to emergency departments and acute medical services at OUH during the second pandemic wave (Oct 1, 2020, to March 6, 2021; figure 1A, appendix p 2). To assess performance across calibrations, we did the analysis with thresholds set during training to achieve sensitivities of 80% and 90%. Additionally, we did a sensitivity analysis to assess susceptibility to imputation strategy, training the models with three independent imputation methods (median, mean, and age-based mean) and examining variance in performance during evaluation. Calibration was assessed by standard calibration curve analysis, with predictions grouped with quantile-based binning.

### External validation at independent NHS trusts

We externally validated CURIAL-Rapide and CURIAL-Lab, calibrated during training to 90% sensitivity, for emergency admissions across three independent UK NHS hospital trusts by comparing model predictions with results of confirmatory molecular testing (SARS-CoV-2 laboratory PCR and the point-of-care PCR devices SAMBA-II and Panther). Participating trusts were the University Hospitals Birmingham NHS Foundation Trust (UHB), Bedfordshire Hospitals NHS Foundation Trust (BH), and Portsmouth Hospitals University NHS Trust (PUH), serving a total population of about 3·5 million. Cycle threshold (Ct) cutoff values for a positive molecular test result adopted local hospital standards. We evaluated the models for all patients aged 18 years or older who had an emergency admission through emergency or acute medical pathways and underwent a blood draw on arrival during specified date ranges (shown in figure 1A). Screening against eligibility criteria, followed by anonymisation, was done by the respective NHS trusts. Patients who did not consent to EHR research, did not receive confirmatory testing for SARS-CoV-2, or had only an invalid confirmatory result with no subsequent valid result were excluded. For trusts where blood gas results were available for electronic extraction, we also evaluated CURIAL-1.0. Evaluation details are shown in figure 1A and in the appendix (pp 8–10).

### Comparison with LFDs

To investigate the suitability of CURIAL-Rapide, CURIAL-Lab, and CURIAL-1·0 as rapid screening tests, we compared sensitivities and NPVs with the Innova SARS-CoV-2 antigen rapid qualitative test (Innova, Pasadena, CA, USA) used within standard-of-care for admissions to OUH during the second pandemic wave (Oct 1, 2020, to March 6, 2021; appendix p 5). From Dec 23, 2020, patients admitted to OUH from acute and emergency care settings (emergency department, ambulatory medical unit, and medical assessment unit) had LFDs done routinely alongside PCR testing. Nasopharyngeal swabs for both tests were collected by trained nursing or medical staff. LFDs were performed in the emergency or acute department and documented in the EHR. Swabs for PCR were transferred to the clinical laboratory in viral transport medium and tested by multiplex PCR (TaqPath, ThermoFisher, Waltham, MA USA), forming the reference standard for evaluation.

### Combined algorithm to enhance the sensitivity of LFD testing

Next, we investigated whether our models could enhance the sensitivity and NPV of LFDs for COVID-19 during emergency admission. We proposed and retrospectively evaluated a novel clinical triage pathway (figure 1C), labelling patients as COVID-19-suspected if they had either a positive CURIAL model (CURIAL-Lab or CURIAL-Rapide) result or a positive LFD result. In our pathway, due to high specificity, patients with positive LFDs could be streamed directly to a COVID-19-positive clinical area, whereas patients with a negative LFD but positive CURIAL result would be managed in an enhanced-precautions area pending PCR adjudication. The pathway aimed to provide enhanced NPVs for patients receiving both negative LFD and CURIAL results, thus reducing the false-negative rate and supporting safe triage directly to a COVID-19-free clinical area. We retrospectively assessed the performance of this novel pathway for all unscheduled admissions to OUH in which patients received LFD and PCR testing, from introduction on Dec 23, 2020, to March 6, 2021 (figure 1A).

### Prospective evaluation of CURIAL-Rapide in a laboratory-free clinical pathway

To prospectively assess the operational and predictive performance of CURIAL-Rapide in a laboratory-free setting, we deployed two OLO rapid haematology analysers in the John Radcliffe Hospital's Emergency Department (Oxford), as part of an OUH-approved service evaluation (Ulysses ID 6907).^28^ We simultaneously aimed to improve routine clinical care by reducing the turnaround time for routine blood test results in the emergency department. The analysis plan and data requirements were determined prospectively and registered with the Trust service evaluation database.

The service evaluation operated from Feb 18, 2021, to May 10, 2021, between 0800 h and 2000 h. Eligible patients were older than 18 years, attending the emergency department with an acute illness and streamed to a bedded clinical area, and had consented to receive FBC analysis and vital signs as part of their care plan. We selected patients allocated to bedded clinical areas because non-ambulatory patients typically have higher acuity, thus being more likely to benefit from faster blood test results and having higher probability of admission. Patients were identified on arrival using the FirstNet system (Cerner Millennium, Cerner, North Kansas City, MO, USA).

Eligible patients were enrolled for additional laboratory-free FBC analysis using OLO, which in conjunction with vital signs was used to generate CURIAL-Rapide predictions. OLO results were uploaded immediately to the EHR, making results available to clinicians and supporting routine care. We excluded patients with an invalid OLO result and no subsequent successful result, thereby ensuring data completeness. Routine SARS-CoV-2 testing was done in line with trust policies, with LFDs done in the department and paired multiplex PCR done on-premises in a dedicated laboratory.

We recorded patients' arrival time to the hospital, measurement time of vital signs, and time-to-results for LFD, PCR, OLO, and laboratory FBC analysis. We also recorded the COVID-19 triage impression of the first-attending junior or senior physician, as documented in the clinical notes using the green–amber–blue categorisation system adopted by Trust policy (green representing a patient whose illness has no features of COVID-19, amber representing an illness with features potentially consistent with COVID-19, and blue representing laboratory-confirmed COVID-19 infection).^29^, ^30^ If the COVID-19 triage category had not been documented by the first-assessing physician, adjudication was done through review of notes using rules-based determination initially by trained medical students and verified by a practicing physician. Patients who had documentation of a new continuous cough, temperature of 37·8°C or higher, or loss or change in sense of smell or taste were adjudicated as an amber (COVID-19 suspected) stream, matching UK Government guidance on the definition of a possible COVID-19 case.^6^ Patients who had PCR-confirmed COVID-19 in the 10 days preceding attendance were adjudicated to the blue (COVID-19 confirmed) stream. Patients with no features of COVID-19 and no documented clinical suspicion were adjudicated to the green stream.

We selected time-to-result as our primary outcome, recognising the role of rapid results in reducing nosocomial transmission. Performance measures were sensitivity, specificity, positive predictive value, and NPV for CURIAL-Rapide and LFDs, and area under receiver operating characteristic curve (AUROC) for CURIAL-Rapide assessed against PCR results. Additional details are provided in the appendix (pp 10–12).

### Statistical analysis

Model training followed a previously described protocol, controlling for age, gender, and ethnicity during training.^5^ We queried relative feature importances, and we calculated SHAP (Shapley Additive Explanations) scores to understand the effects of individual predictors on model predictions. All predictions were generated by application to results of testing from first blood draw and vital signs and compared with confirmatory SARS-CoV-2 genome testing. We report model performances with sensitivity, specificity, positive predictive value, NPV, AUROC, and *F1* score. We computed 95% CIs for sensitivity, specificity, and predictive values using Wilson's Method^31^ and for AUROC using DeLong's method.^32^

During prospective and external evaluation, we calculated and reported the proportions of missing data. Missing data were imputed as the training population median values. Patients with missing PCR results did not meet inclusion criteria and were excluded. To assess for biases by ethnicity, gender, and clinical severity, we did subgroup analyses in which group size was 15 patients or more or 0·25% of the evaluation population. Comparison between model performance alone and the integrated clinical pathway for each model (figure 1C) was done with McNemar's χ^2^.

We estimated a suitable review point for the CURIAL-Rapide and OLO deployment using Buderer's standard formulas.^33^ Predicting a sensitivity of 80% (matching model calibration), specificity of 75%, and prevalence of COVID-19 at 15% among patients in the emergency department, a minimum sample size of 410 enrolled patients was estimated to determine sensitivity and 85 patients to determine specificity (95% confidence and 10% precision).^34^ Therefore, we planned to review model performance once 500 patients had been enrolled to allow for missing or invalid confirmatory tests.

Binary CURIAL-Rapide results (COVID-19 suspected and COVID-19 negative) were generated using OLO results and vital signs. Availability time for CURIAL-Rapide was the later of OLO result time and vital signs recording time because both are required to generate a prediction. No imputation was done because the design ensured data completeness. CURIAL-Rapide predictions were not made available to the attending physician so as not to influence the clinical triage category or decisions to proceed to confirmatory testing for patients being discharged. The time-to-result for PCR, LFD, and CURIAL-Rapide tests were calculated as the time from a patient's first arrival in the emergency department to the time of a test result being available, thus including sample acquisition time. Performance was assessed against a PCR reference standard.

For paired samples, time-to-result was compared between tests by one-tailed Wilcoxon Signed Rank test. We used Kaplan-Meier survival analysis with logrank testing to compare time-to-result by test type. Patients who did not have confirmatory testing at 72 h were excluded as missingness probably represented omission. Mean (SD) are presented for normally distributed data, and median (IQR) for data with a skewed distribution. The software used in this study is described in the appendix (pp 4, 12).

### Role of the funding source

The funders of the study had no role in study design, data collection, data analysis, data interpretation, or writing of the manuscript.

## Results

Our updated training set comprised 114 957 emergency presentations prior to the global COVID-19 outbreak (Dec 1, 2017, to Nov 30, 2019), considered as COVID-19 free, and 701 patient presentations during the first UK pandemic wave with a positive SARS-CoV-2 PCR test (Dec 1, 2019, to June 30, 2021; appendix p 5). Similar to our previous findings, relative feature importance analysis showed that granulocyte counts (basophils and eosinophils), CRP, and oxygen requirements remained the highest-ranking features for CURIAL-1.0 (appendix p 13). SHAP analysis confirmed that CRP and granulocyte counts had the greatest influence on model predictions. Owing to removal of features that were weakly informative, predictor importances were similar between CURIAL-1.0 and CURIAL-Lab; however, granulocyte counts and respiratory rate had greater relative importance in CURIAL-Rapide, reflecting the reduced predictor set.

Between Dec 1, 2019, and March 31, 2021, 72 223 patients were included across four validation cohorts, of whom 4600 had a positive confirmatory test for SARS-CoV-2 (appendix pp 3–10). Patients admitted to PUH and BH trusts had similar ages (69 years, IQR 34, for PUH and 68 years, 34, for BH; Kruskal-Walls p=0·94), whereas patients admitted to UHB were younger (63 years, 37, p<0·0001 for both; table 1). A higher proportion of patients admitted to UHB were women (5462 [53·1%] of 10 293) than those admitted to PUH (17 054 [45·0%] of 37 896) and BH (549 [46·7%] of 1177; χ^2^ p<0·0001) and reported being of South Asian ethnicity (1357 [13·2%] UHB *vs* 170 [0·5%] PUH and 71 (6·0%) BH; χ^2^ p<0·0001).

**Table 1:** Summary population characteristics

		**Training–OUH (pre-pandemic and first wave cases, to 30 June 2020)**		**Prospective validation–OUH**	**External validation (admissions)**			**LFD evaluation–OUH (second wave admissions)**	**Laboratory-free deployment–JRH emergency department**
					Portsmouth Hospitals University NHS Trust	University Hospitals Birmingham NHS Foundation Trust	Bedfordshire Hospitals NHS Foundation Trust		
Cohort		Pre-pandemic	COVID-19 cases	Oct 1, 2020, to March 6, 2021	March 1, 2020, to Feb 28, 2021	Dec 1, 2019, to Oct 29, 2020	Jan 1, 2021, to March 31, 2021	Dec 23, 2020, to March 6, 2021	Feb 18, 2021, to May, 10, 2021
Patients		114 957	701	22 857	37 896	10 293	1177	3207	520
Positive COVID-19 PCR or genome test		0	701	2012 (8·8%)	2005 (5·3%)	439 (4·3%)	144 (12·2%)	355 (11·1%)	10 (2·3%)
Sex									
	Female	61 587 (53·6%)	325 (46·4%)	11 448 (50·1%)	17 054 (45·0%)	5462 (53·1%)	549 (46·6%)	1621 (50·5%)	289 (55·9%)
	Male	53 370 (46·4%)	376 (53·6%)	11 409 (49·9%)	20 839 (55·0%)	4831 (46·9%)	627 (53·3%)	1586 (49·5%)	231 (44·4%)
Age, years		60 (38–76)	72 (55–82)	67 (49–80)	69 (48–82)	63 (42–79)	68 (48–82)	70 (51–82)	76 (60–85)
Positive LFD		..	..	..	..	..	..	207 (6·5%)	≤10
Ethnicity									
	White	93 921 (81·7%)	480 (68·5%)	17 387 (76·1%)	28 704 (75·7%)	6848 (66·5%)	1024 (87·0%)	2491 (77·7%)	419 (80·6%)
	Not stated	13 602 (11·8%)	128 (18·3%)	4127 (18·1%)	8389 (22·1%)	1061 (10·3%)	≤10	513 (16·0%)	80 (15·4%)
	South Asian	2754 (2·4%)	22 (3·1%)	441 (1·9%)	170 (0·4%)	1357 (13·2%)	71 (6·0%)	65 (2·0%)	≤10
	Chinese	284 (0·2%)	*	51 (0·2%)	42 (0·1%)	41 (0·4%)	≤10	*	≤10
	Black	1418 (1·2%)	25 (3·6%)	279 (1·2%)	187 (0·5%)	484 (4·7%)	36 (3·1%)	45 (1·4%)	≤10
	Other	1840 (1·6%)	34 (4·9%)*	410 (1·8%)	269 (0·7%)	333 (3·2%)	29 (2·5%)	72 (2·2%)*	≤10
	Mixed	1138 (1·0%)	12 (1·7%)	162 (0·7%)	135 (0·4%)	169 (1·6%)	13 (1·1%)	21 (0·7%)	≤10

Data are n, n (%), or median (IQR), unless otherwise specified. Population characteristics for OUH pre-pandemic and COVID-19-cases training cohorts, prospective validation cohort of patients attending OUH during the second wave of the UK COVID-19 epidemic, independent validation cohorts of patients admitted to three independent NHS Trusts, admissions to OUH during the second wave receiving LFD testing, and patients enrolled to the CURIAL-Rapide–OLO laboratory-free service evaluation at JRH. The derivation of OUH cohorts is shown in the appendix (p 5). Some n values of ten or lower are not given with precision to preserve deidentification. JRH=John Radcliffe Hospital. LFD=antigen testing with lateral flow device. NHS=National Health Service. OUH=Oxford University Hospitals.

* Indicates merging for statistical disclosure control.

COVID-19 prevalence was highest in the BH cohort owing to the evaluation timeline spanning the second UK pandemic wave (11·1% *vs* 5·3% in PUH and 4·3% in UHB; Fisher's exact test p<0·0001 for both). FBC testing, required by CURIAL-Rapide, was near-ubiquitously done (>98·5%). Feature summaries and data completeness are reported in the appendix (pp 5–8).

For the prospective evaluation of CURIAL-Lab and CURIAL-Rapide, we included 22 857 patients attending OUH emergency and acute medical services during the second pandemic wave who met inclusion criteria, with 2012 testing positive (8·8%; appendix p 6). At the 80% sensitivity configuration, CURIAL-Lab performed similarly to CURIAL-1.0 (sensitivities of 72·9% for CURIAL-Lab and 73·6% for CURIAL-1.0, and specificities of 87·3% for CURIAL-Lab and 86·6% for CURIAL-1.0; McNemar χ^2^ p=0·082), but better than CURIAL-Rapide (sensitivity 74·7% and specificity 78·6%; p<0·0001), representing a trade-off between time-to-result and performance. Both models achieved high NPV (>98%) across 80% and 90% sensitivity configurations and high AUROCs (0·843 for CURIAL-Rapide and 0·878 for CURIAL-Lab, appendix p 8).

We assessed sensitivity to imputation strategy, finding performance stability across multiple imputations (appendix p 8). Therefore, we selected a single imputation strategy for subsequent evaluation (population median). Assessing performance across clinically relevant severity subgroups, we found that the models were equally sensitive for patients discharged from the emergency department as patients admitted to hospital wards outside of the intensive care unit (ICU). However, sensitivity was marginally superior for patients requiring ICU admission (appendix pp 16–17). Calibration curve analyses showed that all models were well calibrated, and that calibration was equivalent across models (appendix p 14).

**Figure 2:**
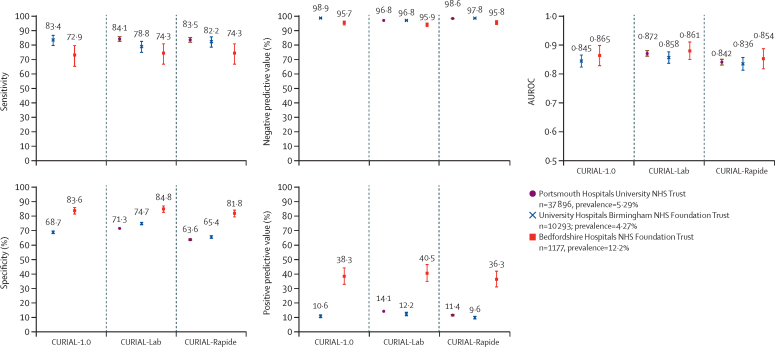
Performance of CURIAL-1.0, CURIAL-Lab, and CURIAL-Rapide during external validation at three independent UK hospitals trusts All models were calibrated during training to achieve 90% sensitivity. Error bars show 95% CIs. Numerical results are shown in the appendix (pp 9–10). AUROC=area under receiver operating characteristic curve. NHS=National Health Service.

In the external validation of CURIAL-Rapide, CURIAL-Lab, and CURIAL-1.0, 49 366 admissions across three external hospital groups met inclusion criteria (figure 2). Performance was consistent across trusts, with CURIAL-Lab achieving marginally higher performance (AUROC range 0·858–0·881, 95% CI range 0·838–0·912) than that of CURIAL-Rapide (0·836–0·854, 0·814–0·889). The sensitivity of both models was higher when applied at PUH (84·1%, 95% CI 82·5–85·7, for CURIAL-Lab and 83·5%, 81·8–85·1 for CURIAL-Rapide) than at BH (74·3%, 66·6–80·7, for both) at the expense of specificity (PUH 71·3%, 70·9–71·8, for CURIAL-Lab and 63·6%, 63·1–64·1, for CURIAL-Rapide *vs* BH 84·8%, 82·5–86·9, for CURIAL-Lab and 81·8%, 79·3–84·0, for CURIAL-Rapide; appendix pp 9–10), possibly reflecting differences in confirmatory testing methods at BH (SAMBA-II and Panther).

Both CURIAL-Rapide and CURIAL-Lab consistently achieved high NPVs, with the highest at UHB (98·8%, 95% CI 98·5–99·0, for both; prevalence 4·27%) and similar at PUH (98·6%, 98·4–98·7, for CURIAL-Rapide and 98·8%, 98·6–98·9 for CURIAL-Lab; prevalence 5·29%). External validation of CURIAL-1.0 at OUH and BH, where blood gas results were available, showed that performance was similar to that of CURIAL-Lab (appendix pp 9–10).

To assess for biases by gender or ethnic group, we did subgroup analyses at each externally validating site (appendix pp 14–16). Rates of misclassification were similar between men and women, for both CURIAL-Lab and CURIAL-Rapide, at UHB (Fishers' exact test p=0·73 for CURIAL-Lab and p=0·66 for CURIAL-Rapide) and BH (p=0·35 for CURIAL-Lab and p=0·66 for CURIAL-Rapide). Women were less likely than men to be misclassified by CURIAL-Lab at PUH (p<0·0001) but equally likely with CURIAL-Rapide (p=0·23). Using CURIAL-Rapide, patients recorded as being of South Asian, Black, or mixed ethnic groups were equally likely to be misclassified as patients from White ethnic groups at BH (p=0·76 for South Asian and p=1·00 for Black and mixed ethnic groups) and PUH (p=0·14 for South Asian, p=0·36 for Black, and p=0·33 for mixed ethnic groups), but were less likely to be misclassified at UHB (p<0·0001 for South Asian, p=0·032 for Black, and p=0·043 for mixed ethnic groups). These findings might reflect differences in the proportion of patients whose ethnicity was recorded as not stated, with BH achieving near-completeness in ethnicity recording (>99%), whereas UHB had 10·3% and PUH 22·1% of patients with no stated ethnicity data (table 1).

**Figure 3:**
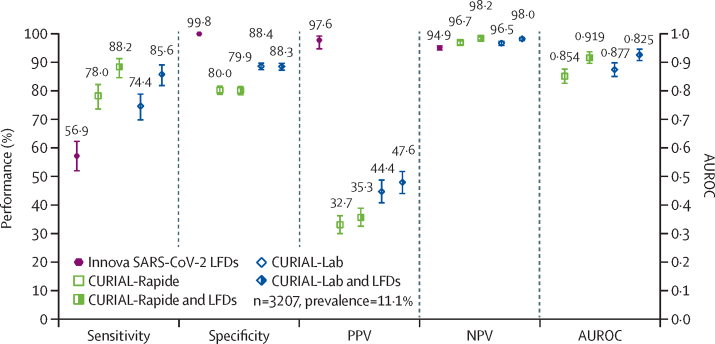
Performance characteristics of Innova SARS-CoV-2 LFD (A), CURIAL-Rapide and CURIAL-Lab (B) calibrated during training to a sensitivity of 80%, and combined clinical pathways (C) Combined clinical pathways consider either a positive CURIAL model (CURIAL-Rapide or CURIAL-Lab) result or a positive LFD test as a COVID-19 suspected case, at Oxford University Hospitals National Health Service Foundation Trust between Dec 23, 2020, and March 6, 2021. Error bars show 95% CIs. Numerical results are shown in the appendix (p 10). AUROC=area under receiver operating characteristic curve. LFD=antigen testing with lateral flow device. NPV=negative predictive value. PPV=positive predictive value.

To compare CURIAL triage performance with lateral flow testing, we applied CURIAL-Rapide and CURIAL-Lab to the first-performed blood tests and vital signs of 3207 patients admitted to OUH and receiving LFD testing (figure 3, appendix p 5). One patient with an invalid result was excluded. The sensitivity of LFDs was 56·9% (51·7–62·0), and specificity was 99·8% (99·6–99·9; appendix p 10).

CURIAL-Rapide and CURIAL-Lab were significantly more sensitive (78·0%, 73·4–82·0, for CURIAL-Rapide and 74·4%, 69·6–78·6, for CURIAL-Lab) than LFDs, and thus achieved higher NPVs (96·7%, 95·9–97·3, for CURIAL-Rapide and 96·5%, 95·7–97·2, for CURIAL-Lab *vs* 94·9%, 94·1–95·6, for LFDs). By contrast, the models were less specific than LFDs (80·0%, 78·5–81·4, for CURIAL-Rapide and 88·4%, 87·2–89·5, for CURIAL-Lab), thereby more safely excluding COVID-19 but at the expense of the false positive rate.

Integrating positive LFD results with CURIAL-Rapide and CURIAL-Lab, forming a combined clinical pathway (figure 1C), significantly improved triage sensitivity (to 88·2%, 84·4–91·1, for CURIAL-Rapide and 85·6%, 81·6–88·9, for CURIAL-Lab) and NPVs (to 98·2%, 97·6–98·7, for CURIAL-Rapide and 98·0%, 97·4–98·5, for CURIAL-Lab), thereby reducing COVID-19 status misclassification (McNemar's χ^2^ p=0·0003 for CURIAL-Rapide, p=0·0004 for CURIAL-Lab). AUROC was significantly improved to 0·919 (0·899–0·940) for a CURIAL-Rapide and LFD pathway, and to 0·925 (0·905–0·945) for a CURIAL-Lab and LFD pathway. CURIAL-Lab performed similarly to CURIAL-1·0 (p=0·86; appendix p 10).

For the deployment and operational evaluation of CURIAL-Rapide at OUH, 520 patients were enrolled to the OLO–CURIAL-Rapide service improvement between Feb 18 and May 10, 2021 (table 1). 436 patients received confirmatory PCR testing within routine care, and ten returned positive results (2·3%). This reflected the falling COVID-19 prevalence due to governmental restrictions and the UK vaccination programme.^35^ 348 patients received LFDs within routine care, with four positive results. Two patients with indeterminate PCR results were excluded from analysis, although both had a negative LFD result and were triaged by the assessing physician to a COVID-19-free clinical pathway. No adverse events were observed. A summary of OLO results and vital signs is shown in the appendix (pp 5–6).

**Figure 4:**
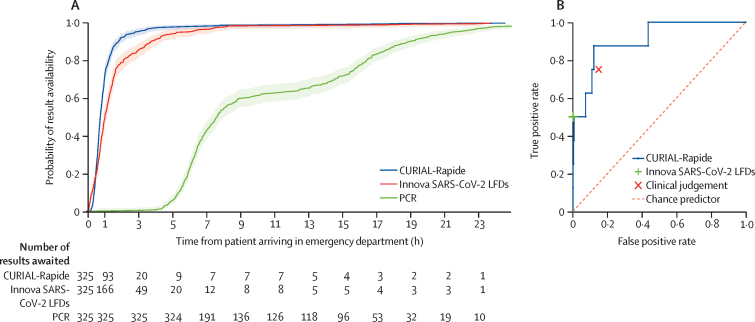
Time-to-result from patient arrival in the emergency department (A) and performance against a PCR standard (B) (A) Kaplan-Meier plots of time-to-result in h from patient arrival in the emergency department for CURIAL-Rapide, Innova SARS-CoV-2 LFD, and PCR swabs tests, alongside number of results awaited, CURIAL-Rapide results that were available sooner than LFD testing (log rank test, p<0·0001), and PCR test results (p<0·0001). (B) Receiver operating characteristic curve showing performance of CURIAL-Rapide, clinical triage done by the first-attending physician, and Innova SARS-CoV-2 LFD, against a PCR reference standard. LFD=antigen testing with lateral flow device.

Median time from registration in the emergency department to CURIAL-Rapide result was 45 min (IQR 32–64), 16 min (26·3%) sooner than for LFDs (Wilcoxon Signed Rank p<0·0001), and 6 h 52 min (90·2%) sooner than for RT-PCR results (p<0·0001; table 2). Kaplan-Meier survival analyses (figure 4A) showed that CURIAL-Rapide results were available sooner than LFD results (log-rank test, p<0·0001) and PCR results (p<0·0001). The median time-to-result for FBC was shorter with OLO (44 min, IQR 31–63) than with laboratory analysis (76 min, 58–100; p<0·0001), showing improvement to routine care.

**Table 2:** Operational and performance characteristics of CURIAL-Rapide, Innova SARS-CoV-2 rapid antigen testing, and clinical triage by the first-attending physician calculated against laboratory RT-PCR testing during OLO-CURIAL-Rapide service evaluation

	**CURIAL-Rapide version 1.0**	**Innova SARS-CoV-2 rapid antigen testing**	**First-attending physician triage**	**Laboratory RT-PCR**
Time from patient arrival in emergency department to result	45 min (32–64)	61 min (36 min 45 s to 99 min)	..	7 h 37 min (06 h 5 min to 15 h 39 min)
Sensitivity	87·5% (52·9–97·8)	50·0% (21·5–78·5)	75·0% (40·9–92·9)	..
Specificity	85·4% (81·3–88·7)	100% (98·9–100·0)	85·1% (81·0–88·4)	..
Accuracy	85·4% (81·4–88·7)	98·9% (97·2–99·6)	84·9% (80·8–88·2)	..
Positive predictive value	11·9% (5·9–22·5)	100% (51·0–100·0)	10·2% (4·7–20·5)	..
Negative predictive value	99·7% (98·2–99·9)	98·9% (97·2–99·6)	99·3% (97·6–99·8)	..
AUROC	0·907 (0·803–1·000)	..	..	..

Data are median (IQR) or measure (95% CI). AUROC=area under receiver operating characteristic curve.

CURIAL-Rapide results had a NPV of 99·7% (95% CI 98·2–99·9), specificity of 85·4% (81·3–88·7), and AUROC of 0·907 (0·803–1·00; table 2). The point estimate of CURIAL-Rapide's sensitivity was 87·5%, however 95% CIs were wide owing to the lower-than-expected prevalence of COVID-19 (52·9–97·8).

In one presentation, a patient given a negative CURIAL-Rapide prediction went on to have a positive SARS-CoV-2 PCR test. However, the patient had a negative LFD result, did not have COVID-19 symptoms, and was triaged to a COVID-19-free clinical area. We noted that the individual had also been enrolled to the service evaluation 10 days before; on that occasion, they had a positive CURIAL-Rapide prediction and positive LFD and PCR tests. This case raises the possibility of a latent positive PCR result, detecting non-infectious residual viral fragments, on the date of the second presentation.^36^

Rates of COVID-19 status misclassification were similar between CURIAL-Rapide and physician judgment (McNemar's Exact test p=0·91). Moreover, of the 53 patients who were triaged to a COVID-19-suspected (amber) pathway by the attending physician but went on to test negative by PCR, 31 (58·5%) had a negative CURIAL-Rapide prediction, showing that the artificial intelligence system could reduce operational strain by expediting exclusion of infection.

## Discussion

National health policy recognises that effective in-hospital triage is necessary to safeguard patient and staff safety during a pandemic. However, logistical and performance limitations of current testing options for SARS-CoV-2 contribute to treatment delays and operational strain, despite improvements in turnaround time. Although many hospitals have adopted LFDs for emergency admissions, we found a high false negative rate of 43 missed cases per 100 PCR-positive admissions in this context (figure 3), highlighting a need for more sensitive solutions.^37^

In this study, we did a multicentre validation and prospective evaluation of our artificial intelligence screening models in a real-world emergency care setting. During validation, CURIAL-Lab and CURIAL-Rapide performed consistently well across four hospital groups, with high negative predictive performance supporting their use to safely stream patients to COVID-19-free clinical areas. When combined with LFDs, our models' superior NPVs improved triage sensitivity, thereby reducing missed COVID-19 cases by 72% with CURIAL-Rapide (from 43 missed cases per 100 PCR-positive admissions to 12 per 100). In prospective deployment, CURIAL-Rapide achieved the fastest result-time to date for artificial intelligence-driven COVID-19 screening in a hospital emergency department, using laboratory-free haematology analysis to provide results 45 min from patients' first arrival to the emergency department, and 26·3% faster than LFD results. Moreover, classification performance for CURIAL-Rapide was high. Together, our findings show the generalisability, efficacy, and real-world operational benefits of artificial intelligence-driven screening for COVID-19 in emergency care.

The strengths of our study include the extensiveness of our model validation, performed for more than 72 000 patients across four hospital groups. The study populations included geographical spread, across three UK regions, and temporal spread, spanning the full length of the pandemic and thus including vaccinated patients, patients with SARS-CoV-2 variants, and the full observed range of prevalences. Another substantial strength was the use of external cohorts containing all unscheduled adult admissions, representing the populations of intended use and being easily definable and reproducible, thereby addressing sector-wide concerns of unrepresentative validation.^25^, ^26^ Recognising concerns of bias, we analysed performance across subgroups by gender and ethnicity, finding that no individual group had consistently better or worse performance across the three external sites. Moreover, sensitivity was similar for patients discharged from the emergency department compared with those admitted to hospital wards (non-ICU), suggesting that patients attending the emergency department with lower COVID-19 severity were not at higher risk of a false negative result. As levels of predictor availability were high (>98·5% for FBC), we found that the models were widely applicable and not sensitive to imputation method for missing data.

To our knowledge, our study is the first to investigate and validate an emergency care pathway using an artificial intelligence test in parallel with LFDs, quantitatively assessing the improvements to sensitivity over current practice. Patients receiving a negative LFD and CURIAL result represent an important beneficiary population and would be streamed directly to COVID-19-free clinical areas upon receiving results of the two tests, which are available much sooner than PCR results. Additionally, the pathway allows for prioritisation of rapid PCR testing capacity, where available but limited in supply, by identifying an enriched subpopulation with greater risk of a positive SARS-CoV-2 confirmatory test. Translational advantages of the combined approach include the potential for low-risk clinical deployment, with the pathway design ensuring that performance is at least equal to the use of LFDs alone.

An important strength of the CURIAL-Rapide deployment was its real-world setting and operational focus, prospectively evaluating time-to-result from arrival in the emergency department alongside performance metrics. We considered physicians' impressions, finding that CURIAL-Rapide correctly excluded infection for 58·5% of patients who were clinically triaged to a COVID-19-suspected area but went on to test negative by PCR. This evaluation provides evidence for the operational benefits of the artificial intelligence, reducing delays in transfers to wards by way of faster results and potentially reducing the numbers of patients awaiting PCR results in enhanced-precautions areas. Moreover, CURIAL-Rapide's laboratory-free approach can support time-crucial decision making and triage in remote care settings where laboratory facilities are less accessible.

Notable limitations of the validation include that it was solely UK based, and confirmatory testing protocol might have varied between laboratories. We considered the possibility that confirmatory results at higher Ct values might represent latent positive results, thereby penalising the apparent sensitivity of the CURIAL models; however, Ct values are not directly comparable between assays, and there is limited consensus on the threshold at which patients are no longer considered infectious.^36^ Therefore, we selected the locally adopted Ct cutoff for reporting positive clinical results, reflecting majority practice and safeguarding against optimistic performance reporting. We were unable to quantify the number of patients who were vaccinated as we could not link our deidentified hospital datasets with vaccination records.

The limitations of the CURIAL-Rapide deployment were that, although the a-priori enrolment target was achieved, the desired precision for sensitivity was not achieved due to falling disease prevalence associated with the UK vaccination programme and public health measures.^38^ However, the evaluation was sufficient to determine specificity, AUROC, and negative predictive value. As a service improvement, we used a convenience series, limiting OLO operation to daytime and evening hours (0800 h to 2000 h) for logistical reasons and enrolling only patients streamed to bedded areas who were more likely to benefit from rapid FBC testing. Moreover, although LFD testing was hospital policy, 33% of enrolled patients did not have a coded result in the EHR, raising the possibility that these might have been recorded incorrectly.

In conclusion, the CURIAL solutions effectively screen patients requiring emergency admission for COVID-19. Strengths of the approach are its potential for rapid scale and near-universal applicability, requiring only routine tests done within 1 h of presenting to existing hospital care pathways. Consequently, CURIAL-Lab screening entails negligible additional cost and might permit reduction of routine PCR testing owing to high NPVs. By contrast, competing approaches require additional testing and are thus limited in applicability, have longer result-time, and entail higher costs.^39^ Where faster results are desirable, the CURIAL-Rapide approach eliminates the need for blood sample transportation and laboratory processing by use of a well established, robust, and affordable point-of-care test (OLO) already in clinical use across the UK (approximate inclusive cost of about £9 per sample [about US$12·50]). The CURIAL models are extensively validated by use of pragmatic, real-world emergency department populations, across multiple hospitals, and showed no consistent biases by gender or ethnic groups.

Our work shows generalisability, efficacy, and real-world operational benefits of artificial intelligence-driven screening for COVID-19 in emergency care. Future work would assess international generalisability, evaluate clinician–model interactions, and assess sensitivity of model performance across vaccination types and infection with SARS-CoV-2 variants of concern. Learnings from the ongoing deployment of artificial intelligence systems into front-line care would guide subsequent translational strategies and identify barriers to their sustained adoption.^40^

## Data sharing

Data from OUH studied here are available from the Infections in Oxfordshire Research Database (https://oxfordbrc.nihr.ac.uk/research-themes-overview/antimicrobial-resistance-and-modernising-microbiology/infections-in-oxfordshire-research-database-iord/), subject to an application meeting the ethical and governance requirements of the database. Data from UHB, PUH, and BH are available on reasonable request to the respective trusts, subject to NHS Health Research Authority requirements. The validation study code is available at https://github.com/andrewsoltan/CURIAL-Validation.

## Declaration of interests

DWE reports personal fees from Gilead, outside the submitted work. DAC reports personal fees from Oxford University Innovation, BioBeats, and Sensyne Health; and participation on a data safety monitoring board or advisory board for Bristol Myers Squibb, outside the submitted work. All other authors declare no competing interests.
