# Supplementary material for: Real-world evaluation of rapid and laboratory-free COVID-19 triage for emergency care: external validation and pilot deployment of artificial intelligence driven screening
Source: Lancet Digit Health. 2022 Mar 9;4(4):e266–78. doi: 10.1016/S2589-7500(21)00272-7 (PMC8906813; doi:10.1016/S2589-7500(21)00272-7)
Supplement: Supplementary appendix [file mmc1.pdf]

THE LANCET
Digital Health

Supplementary appendix

This appendix formed part of the original submission and has been peer reviewed. We post it as supplied by the authors.

Supplement to: Soltan AAS, Yang J, Pattanshetty R, et al. Real-world evaluation of rapid and laboratory-free COVID-19 triage for emergency care: external validation and pilot deployment of artificial intelligence driven screening. *Lancet Digit Health* 2022; published online March 9. https://doi.org/10.1016/S2589-7500(21)00272-7.

Supplementary Material

Real-world evaluation of rapid and laboratory-free COVID-19 triage for emergency care: external validation and pilot deployment of artificial intelligence driven screening AAS Soltan et al. 2022

Appendix A

CURIAL Translational Collaborative

We thank all healthcare professionals and students who have supported the CURIAL-Rapide & OLO service evaluation (Oxford University Hospitals NHS Foundation Trust & University of Oxford, Oxford, UK):

Adam Watson
Akshay Bhargav
Alex Tough
Alice Rogers
Ayisha M.A. Shaikh
Carolina Valensise
Charlotte Lee
Claire Otasowie
David Metcalfe
Ekta Agarwal
Elham Zareh
Evelyn Thangaraj
Florence Pickles
Gabriella Kelly
Gayatri Tadikamalla
George Shaw
Heather Tong
Hettie Davies
Jaspreet Bahra
Jessica Morgan
Joe Wilson
Joseph Cutteridge
Katherine O'Byrne
Luiza Farache Trajano
Madeleine Oliver
Maria Pikoula
Maya Mendoza
Melissa Keevil
Muhammad Faisal
Natasha Dole
Oscar Deal
Rebecca Conway-Jones
Shajeel Sattar
Sneha Kundoor
Sumaiyah Shah
Vani Muthusami

Appendix B:

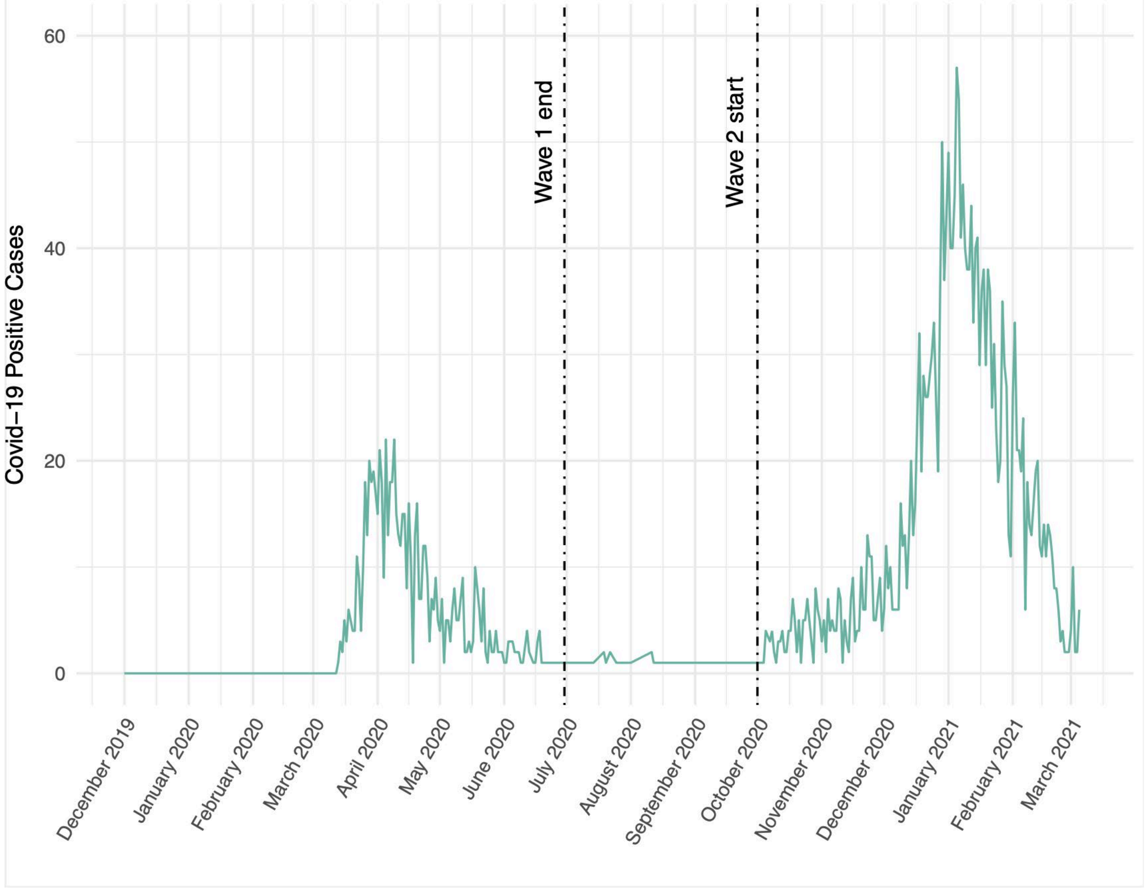

Supplementary Figure S1: Daily number of patients presenting to Oxford University Hospitals NHS Foundation Trust testing positive for COVID-19, between 1st December 2019 and 6th March 2021.

Appendix C:
Model Development: Inclusion & Exclusion Criteria
As previously, we included all patients attending acute and emergency care settings at Oxford University Hospitals NHS foundation trust who received routine blood tests on arrival, considering presentations before December 1, 2019, and thus before the pandemic, as the COVID-19-negative (control) cohort. The model was trained previously using clinical data from patients presenting to emergency and acute medical services at Oxford University Hospitals (OUH) between Dec 1, 2017 and April 19, 2020; additional data on all COVID-19-positive patient presentations to June 30, 2020 were added to encompass the 'first-wave' of the COVID-19 pandemic in the UK. Patients presenting between December 1, 2019 and June 30, 2020 with PCR confirmed SARS-CoV-2 infection formed the COVID-19-positive (cases) cohort. We excluded patients who opted out of electronic health record (EHR) research and those who did not receive laboratory blood tests or were younger than 18 years of age. Due to incomplete penetrance of testing during the first wave of the pandemic, and imperfect sensitivity of the PCR test, there is uncertainty in the viral status of patients presenting during the pandemic who were untested or tested negative. We therefore selected a pre-pandemic control cohort during training to ensure absence of disease in patients labelled as COVID-19-negative.
Clinical features extracted for each presentation included first-performed blood tests, blood gases, vital signs measurements and PCR testing for SARS-CoV-2 (Abbott Architect [Abbott, Maidenhead, UK], TaqPath [Thermo Fisher Scientific, Massachusetts, USA] and Public Health England-designed RNA-dependent RNA polymerase assays). A list of extracted clinical features is shown in Supplementary Table S1.

Missing Data
Multiple imputation strategies, population median, population mean, and age-based imputation, were separately used to impute missing data initially. As a sensitivity analysis to assess for effects of imputation strategy on model performance, we assessed performance of models trained using each imputation method prospectively for all patients attending emergency departments and acute medical services across OUH during the second-wave of the COVID-19 pandemic (October 01, 2020 and March 06, 2021; Figure 1 & Table 1). Mean performance was reported alongside SD in Supplementary Table S6, with narrow standard deviations in all performance metrics demonstrating resilience to imputation method. We therefore subsequently evaluated models trained with missing data imputed using population median, reporting results alongside 95% confidence intervals (CIs).

Model Training & Prospective Evaluation:
We repeated training and optimisation of our eXtreme Gradient BOOSTed tree model (XGBoost) to discriminate COVID-19-positive cases from pre-pandemic COVID-19-negative controls, for each of the three feature-sets (Figure 1B).[1] During training using 'first wave' case, controls were matched for age, gender, and ethnicity at a ratio of 1:20. Thresholds were calibrated to achieve sensitivities of 80% and 90% during training, using stratified 10-fold cross validation.

XGBoost is a generalisation of boosting to an arbitrary differentiable loss function. XGBoost is more robust to outliers and has high predictive power. The scikit-learn

(v0.24), LIBLINEAR (v2.41) and XGBoost (v1.2.0) modules for Python were used during model development and classifier evaluation.

Supplementary Table S1: Clinical parameters forming the data-extraction template from training and externally validating NHS sites

**Clinical Descriptors:**	**Presentation Blood Tests:**	**Presentation Blood Gas:**	**Premorbid Clinical Data**
Study ID	PresentationHAEMOGLOBIN	PresentationPOCT pC02	BaselineHAEMOGLOBIN
Presentation Date	PresentationWHITE CELLS	PresentationPOCT sO2	BaselineWHITE CELLS
Ethnicity	PresentationPLATELETS	PresentationPOCT pO2	BaselinePLATELETS
Age at presentation	PresentationMEAN CELL VOL.	PresentationPCT cBASE(Ecf)c	BaselineMEAN CELL VOL.
Gender (M/F)	PresentationRED CELL COUNT	PresentationPCT CO3(P,st)c	BaselineRED CELL COUNT
Comorbidities (ICD10)	PresentationNEUTROPHILS	PresentationPOCT Hctc	BaselineNEUTROPHILS
Outcome	PresentationHAEMATOCRIT	PresentationPOCT FO2Hb	BaselineHAEMATOCRIT
**Vital Signs:**	PresentationLYMPHOCYTES	PresentationPOCT ctO2c	BaselineLYMPHOCYTES
AdmissionRespRate	PresentationMEAN CELL HGB	PresentationPOCT cGLU	BaselineMEAN CELL HGB
AdmissionHeartRate	PresentationMONOCYTES	PresentationPOCT cK+	BaselineMONOCYTES
AdmissionBloodPressure	PresentationEOSINOPHILS	PresentationPOCT cNA+	BaselineEOSINOPHILS
AdmissionSpO2	PresentationBASOPHILS	PresentationPOCT cLAC	BaselineBASOPHILS
AdmissionOxygenDeliveryDevice	Presentation MCH	PresentationPOCT cCA++	BaselineMEAN CELL HGB CONC
AdmissionTemperature	PresentationMPV		BaselineSODIUM
**Microbiology:**	PresentationNRBC A		BaselineALBUMIN
SARS-CoV-2 PCR	PresentationNRBC %		BaselineALK.PHOSPHATASE
SARS-CoV-2 RESULT TYPE	PresentationSODIUM		BaselineALT
SARS-CoV-2 Antigen Test Result	PresentationALBUMIN		BaselineUREA
INFLUENZAPCR	PresentationALK.PHOSPHATASE		BaselineBILIRUBIN
RespiratoryPCR (Biofire)	PresentationALT		BaselineCREATININE
	PresentationUREA		BaselineeGFR
	PresentationBILIRUBIN		BaselinePOTASSIUM
	PresentationCREATININE		BaselineCALCIUM
	PresentationeGFR		BaselineADJUSTED CALC.
	PresentationPOTASSIUM		BaselineCRP
	PresentationCALCIUM		BaselineProthromb. Time
	PresentationADJUSTED CALC.		BaselineAPTT
	PresentationPHOSPHATE		BaselineINR
	PresentationCRP		BaselinePOCT pC02
	PresentationProthromb. Time		BaselinePOCT sO2
	PresentationPOCT ctHb		BaselinePOCT pO2
	PresentationGLUCOSE		BaselinePCT cBASE(Ecf)c
	PresentationAPTT		BaselinePCT CO3(P,st)c
	PresentationINR		BaselinePOCT Hctc
			BaselinePOCT FO2Hb
			BaselinePOCT ctO2c
			BaselinePOCT Cglu
			BaselinePOCT cK+
			BaselinePOCT cNA+
			BaselinePOCT cLAC
			BaselinePOCT cCA++

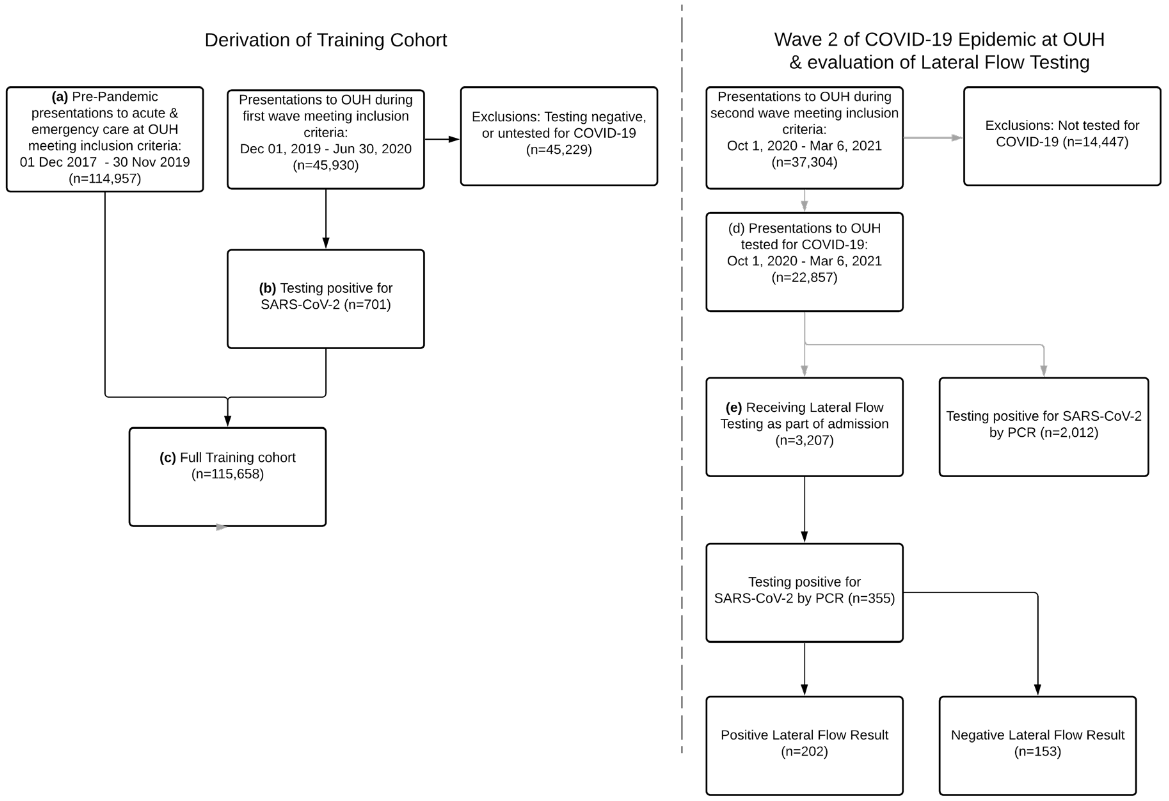

Supplementary Figure S2: Participant flow diagram showing patients attending OUH, who met inclusion and exclusion criteria, for (a) the pre-pandemic training cohort and (b) COVID-19-cases cohort, combining to form (c) a full training cohort for model development. Patients attending OUH during the second wave of the UK COVID-19 epidemic, between Oct 1, 2020 and Mar 6, 2020, meeting inclusion and exclusion criteria, formed (d) the second wave analysis cohort, of which a subset (e) received Lateral Flow Testing within routine care as part of an admission.

Supplementary Table S2: Distribution of vital signs, reported as median and interquartile ranges, for each patient cohort.

	**Training**		**Prospective Test**	**External Validation (Admissions)**			**LFD Evaluation**	**Lab-free Evaluation**
	Oxford University Hospitals (pre-pandemic & wave 1 cases, to 30 June 2020)		Oxford University Hospitals	Portsmouth Hospitals University NHS Trust	University Hospitals Birmingham NHS Foundation Trust	Bedfordshire Hospitals NHS Foundation Trust	Oxford University Hospitals (wave 2 receiving LFDs)	John Radcliffe Hospital ED
	Pre-pandemic cohort	COVID-19-cases cohort	October 1, 2020 – March 6, 2021	March 1, 2020 - February 28, 2021	December 01, 2019 - October 29, 2020	January 1, 2021 - March 31, 2021	December 23, 2020 – March 6, 2021	Feb 18, 2021 – May 10, 2021
**Respiratory Rate (breath/min)**	18.0 (16.0-19.0)	20.0 (18.0-24.0)	18.0 (16.6-19.0)	17.0 (16.0-19.0)	18.0 (17.0-20.0)	18.0 (16.0-20.0)	18.0 (17.0-20.0)	18.0 (17.0-20.0)
**Heart Rate (beats/min)**	82.0 (71.0-96.0)	88.0 (75.0-101.0)	84.0 (72.0-97.0)	82.0 (71.0-95.0)	86.0 (73.0-101.0)	84.0 (73.0-97.0)	87.0 (75.0-101.0)	82.0 (70.0-97.0)
**Systolic Blood Pressure (mmHg)**	132.0 (118.0-150.0)	131.0 (115.0-146.0)	134.0 (119.0-152.0)	128.0 (114.0-146.0)	136.0 (119.0-155.0)	131.0 (116.0-149.0)	136.0 (120.0-156.0)	146.5 (126.0-168.0)
**Diastolic Blood Pressure (mmHg)**	74.0 (65.0-84.0)	74.0 (64.0-84.0)	75.0 (65.0-85.0)	76.0 (67.0-84.0)	77.0 (68.0-87.0)	78.0 (68.0-88.0)	76.0 (65.0-87.0)	79.0 (68.0-90.8)

Oxygen Saturation (%)	97.0 (96.0-99.0)	96.0 (94.0-97.0)	97.0 (96.0-97.9)	97.0 (95.0-98.0)	97.0 (95.0-98.0)	97.0 (96.0-99.0)	97.0 (95.0-97.7)	97.0 (96.0-99.0)
Tympanic Temperature (C)	36.5 (36.1-36.9)	36.9 (36.3-37.6)	36.3 (36.0-36.7)	36.3 (36.0-36.8)	36.7 (36.4-37.2)	36.5 (36.4-36.9)	36.3 (35.9-36.8)	36.3 (35.8-36.8)

Supplementary Table S3: Distribution of blood test features, reported as median and interquartile ranges, for each patient cohort.

	**Training**		**Prospective Test**	**External Validation (Admissions)**			**LFD Evaluation**	**Lab-free Evaluation**
	Oxford University Hospitals (pre-pandemic & wave 1 cases, to 30 June 2020)		Oxford University Hospitals	Portsmouth Hospitals University NHS Trust	University Hospitals Birmingham NHS Foundation Trust	Bedfordshire Hospitals NHS Foundation Trust	Oxford University Hospitals (wave 2 receiving LFDs)	John Radcliffe Hospital ED (OLO FBC results)
	Prepandemic cohort	COVID-19-cases cohort	October 1, 2020 – March 6, 2021	March 1, 2020 - February 28, 2021	December 1, 2019 - October 29, 2020	January 1, 2021 - March 31, 2021	December 23, 2020 – March 6, 2021	Feb 18, 2021 – May 10, 2021
**HAEMOGLOBIN (g/L)**	130.0 (116.0-142.0)	130.0 (114.0-144.0)	129.0 (114.0-142.0)	129.0 (114.0-143.0)	127.0 (113.0-140.0)	134.0 (119.0-146.0)	131.0 (116.0-144.0)	125.0 (112.0-137.5)
**WHITE CELLS (10^9 l^{-1})**	8.45 (6.46-11.18)	6.98 (5.14-9.72)	8.94 (6.7-12.06)	8.6 (6.7-11.3)	9.4 (7.1-12.6)	9.2 (6.9-12.5)	9.43 (7.05-12.73)	8.56 (6.68-11.37)
**PLATELETS (10^9 l^{-1})**	249.0 (199.0-307.0)	215.0 (163.0-283.5)	251.0 (198.0-314.0)	251.0 (199.0-312.0)	247.0 (196.0-311.0)	246.0 (196.0-310.0)	249.0 (195.0-313.0)	223.0 (183.5-270.5)
**MEAN CELL VOL (fl)**	89.6 (86.0-93.4)	90.2 (86.6-94.2)	90.2 (86.6-94.2)	89.0 (84.9-93.0)	89.9 (86.2-93.6)	88.0 (85.0-92.0)	90.0 (86.4-94.3)	90.2 (87.1-93.7)
**NEUTROPHILS (10^9 l^{-1})**	5.72 (3.99-8.36)	5.11 (3.48-7.49)	6.44 (4.4-9.55)	5.9 (4.2-8.6)	6.9 (4.7-10.0)	6.8 (4.7-9.73)	6.97 (4.68-10.19)	6.26 (4.36-9.05)
**HAEMATOCRIT**	0.39 (0.35-0.42)	0.4 (0.35-0.44)	0.39 (0.35-0.43)	0.39 (0.34-0.42)	0.38 (0.34-0.42)	0.39 (0.35-0.43)	0.4 (0.36-0.43)	0.37 (0.33-0.41)
**LYMPHOCYTES (10^9 l^{-1})**	1.51 (1.0-2.13)	0.96 (0.65-1.38)	1.31 (0.85-1.89)	1.5 (0.97-2.2)	1.3 (0.9-1.9)	1.27 (0.86-1.83)	1.26 (0.83-1.89)	1.25 (0.86-1.78)
**MONOCYTES (10^9 l^{-1})**	0.64 (0.48-0.85)	0.49 (0.35-0.74)	0.66 (0.48-0.89)	0.63 (0.48-0.85)	0.7 (0.5-0.9)	0.66 (0.48-0.92)	0.68 (0.49-0.93)	0.59 (0.43-0.78)
**EOSINOPHILS (10^9 l^{-1})**	0.1 (0.04-0.2)	0.01 (0.0-0.06)	0.07 (0.02-0.16)	0.1 (0.02-0.2)	0.1 (0.0-0.2)	0.06 (0.02-0.16)	0.06 (0.01-0.14)	0.09 (0.05-0.17)
**BASOPHILS (10^9 l^{-1})**	0.04 (0.03-0.06)	0.02 (0.01-0.03)	0.04 (0.02-0.06)	0.04 (0.02-0.06)	0.1 (0.0-0.1)	0.05 (0.03-0.07)	0.04 (0.02-0.06)	0.03 (0.01-0.04)
**SODIUM (mM)**	138.0 (136.0-140.0)	136.0 (134.0-139.0)	138.0 (135.0-140.0)	138.0 (136.0-140.0)	137.0 (134.0-139.0)	138.0 (136.0-140.0)	138.0 (135.0-140.0)	
**ALBUMIN (g/L)**	36.0 (32.0-39.0)	32.0 (28.0-35.0)	36.0 (31.0-39.0)	36.0 (31.0-40.0)	36.0 (32.0-40.0)	35.0 (31.0-39.0)	36.0 (31.0-39.0)	
**ALKALINE PHOSPHATASE (IU/L)**	80.0 (64.0-105.0)	82.0 (64.0-108.0)	84.0 (66.0-112.0)	84.0 (67.0-109.0)	90.0 (71.0-119.0)	94.0 (74.5-122.0)	86.0 (69.0-115.0)	
**ALT (IU/L)**	18.0 (13.0-28.0)	25.0 (17.0-41.0)	20.0 (13.0-33.0)	19.0 (13.0-30.0)	19.0 (13.0-30.0)	20.0 (13.0-31.0)	20.0 (13.0-33.0)	
**UREA (mM)**	5.3 (4.0-7.4)	5.9 (4.2-9.07)	5.7 (4.2-8.3)	5.2 (3.8-7.6)	6.2 (4.5-9.0)	5.8 (4.2-8.3)	5.9 (4.3-8.8)	
**BILIRUBIN (umol/L)**	9.0 (6.0-13.0)	9.0 (7.0-13.25)	9.0 (6.0-14.0)	10.0 (7.0-16.0)	10.0 (7.0-15.0)	10.0 (7.0-14.0)	10.0 (7.0-14.0)	
**CREATININE (umol/L)**	73.0 (60.0-93.0)	79.0 (65.0-106.0)	74.0 (60.0-97.0)	74.0 (60.0-96.0)	78.0 (62.0-105.0)	80.5 (65.75-104.0)	74.0 (60.0-98.0)	
**eGFR (ml/min)**	85.0 (63.0-150.0)	78.0 (53.0-150.0)	84.0 (58.0-150.0)	83.0 (60.0-90.0)	76.0 (52.0-90.0)	76.0 (54.0-90.0)	82.0 (56.0-150.0)	
**POTASSIUM (mM)**	4.0 (3.7-4.3)	4.0 (3.7-4.3)	4.0 (3.8-4.4)	4.2 (3.9-4.4)	4.1 (3.8-4.4)	4.3 (4.0-4.6)	4.1 (3.8-4.4)	
**CRP (mg/L)**	8.6 (2.3-39.0)	72.5 (23.8-143.6)	15.8 (3.5-67.4)	13.0 (3.0-71.0)	12.0 (3.0-61.0)	10.7 (2.8-48.78)	17.9 (3.6-77.5)	

Supplementary Table S4: Distribution of blood gas features, reported as median and interquartile ranges for each patient cohort.

	Training		Prospective Test	External Validation (Admissions)		LFD Evaluation
	Oxford University Hospitals (pre-pandemic & wave 1 cases, to 30 June 2020)		Oxford University Hospitals	University Hospitals Birmingham NHS Foundation Trust	Bedfordshire Hospitals NHS Foundation Trust	Oxford University Hospitals (wave 2 receiving LFDs)
	Prepandemic cohort	COVID-19-cases cohort	October 1, 2020 – March 6, 2021	December 01, 2019 - October 29, 2020	January 1, 2021 - March 31, 2021	December 23, 2020 – March 6, 2021
**pCO2 (kPa)**	5.57 (4.94-6.22)	5.34 (4.57-6.01)	5.61 (4.95-6.28)	5.7 (5.0-6.5)	5.72 (5.03-6.43)	5.68 (5.0-6.4)
**O2 Sat (%)**	64.5 (44.0-83.8)	65.15 (38.85-84.68)	65.3 (43.6-85.8)	69.8 (44.6-89.9)	68.0 (44.0-88.9)	60.35 (40.05-80.2)
**pO2 (kPa)**	4.68 (3.51-6.53)	4.62 (3.4-6.7)	4.79 (3.54-6.92)	5.0 (3.4-7.4)	4.86 (3.41-7.2)	4.52 (3.44-6.2)
**BE Std (mM)**	1.3 (-0.7-3.2)	1.3 (-0.8-3.3)	1.4 (-0.8-3.5)	-0.1 (-2.1-1.5)	2.2 (0.02-4.2)	1.6 (-0.6-3.8)
**Bicarbonate (mM)**	24.7 (23.2-26.0)	24.7 (23.2-26.3)	24.8 (23.2-26.3)	24.9 (22.6-27.1)	27.2 (24.72-29.6)	24.8 (23.1-26.3)
**Haematocrit**	41.6 (37.2-45.4)	41.3 (36.8-45.8)	41.3 (36.4-45.5)	43.0 (38.7-46.5)	41.8 (36.9-45.9)	42.0 (37.3-46.0)
**Glucose (mM)**	6.2 (5.4-7.5)	6.6 (5.7-8.2)	6.4 (5.5-8.0)	6.73 (5.81-8.48)	6.4 (5.5-8.0)	6.6 (5.6-8.3)
**K+ (mM)**	3.9 (3.7-4.3)	3.85 (3.6-4.2)	4.0 (3.7-4.3)	3.96 (3.66-4.3)	4.0 (3.7-4.3)	4.0 (3.7-4.3)
**Na+ (mM)**	138.0 (135.0-141.0)	137.0 (133.0-140.0)	138.0 (135.0-141.0)	140.0 (137.2-141.9)	139.0 (135.0-141.0)	138.0 (135.0-141.0)
**cLAC (mM)**	1.3 (0.9-1.9)	1.4 (1.24-2.0)	1.4 (1.24-1.9)	1.64 (1.25-2.27)	1.3 (1.0-1.9)	1.4 (1.24-2.1)
**Ca2+ (mM)**	1.18 (1.14-1.21)	1.12 (1.08-1.16)	1.17 (1.13-1.21)	1.21 (1.16-1.24)	1.17 (1.12-1.2)	1.17 (1.14-1.21)
**Haemoglobin (g/dL)**	136.0 (121.0-148.0)	134.0 (120.0-149.0)	135.0 (118.0-148.0)	133.1 (118.4-146.3)	136.0 (120.0-150.0)	137.0 (121.0-150.0)

Supplementary Table S5: Numbers of participants with data-completeness for each predictor, across each evaluation cohort.

	Prospective Test	External Validation (Admissions)			LFD Evaluation	Lab-free Evaluation
	Oxford University Hospitals	Bedfordshire Hospitals NHS Foundation Trust	University Hospitals Birmingham NHS Foundation Trust	Portsmouth Hospitals University NHS Trust	Oxford University Hospitals (wave 2 receiving LFDs)	John Radcliffe Hospital ED (OLO FBC)
	October 1, 2020 – March 6, 2021	January 1, 2021 - March 31, 2021	December 1, 2019 - October 29, 2020	March 1, 2020 - February 28, 2021	December 23, 2020 – March 6, 2021	Feb 18, 2021 – May 10, 2021
HAEMOGLOBIN (g/L)	22532/22857 (98.6%)	10243/10293 (99.5%)	37761/37896 (99.6%)	1177/1177 (100.0%)	3175/3207 (99.0%)	520/520 (100%)
WHITE CELLS (10^9 l^{-1})	22532/22857 (98.6%)	10244/10293 (99.5%)	37756/37896 (99.6%)	1177/1177 (100.0%)	3175/3207 (99.0%)	520/520 (100%)
PLATELETS (10^9 l^{-1})	22511/22857 (98.5%)	10230/10293 (99.4%)	37719/37896 (99.5%)	1172/1177 (99.6%)	3173/3207 (98.9%)	520/520 (100%)
MEAN CELL VOL (fl)	22532/22857 (98.6%)	10288/10293 (100.0%)	37750/37896 (99.6%)	1177/1177 (100.0%)	3175/3207 (99.0%)	520/520 (100%)
NEUTROPHILS (10^9 l^{-1})	22417/22857 (98.1%)	10277/10293 (99.8%)	37734/37896 (99.6%)	1177/1177 (100.0%)	3163/3207 (98.6%)	520/520 (100%)
HAEMATOCRIT	22532/22857 (98.6%)	10288/10293 (100.0%)	37755/37896 (99.6%)	1177/1177 (100.0%)	3175/3207 (99.0%)	520/520 (100%)
LYMPHOCYTES (10^9 l^{-1})	22430/22857 (98.1%)	10274/10293 (99.8%)	37736/37896 (99.6%)	1177/1177 (100.0%)	3163/3207 (98.6%)	520/520 (100%)
MONOCYTES (10^9 l^{-1})	22452/22857 (98.2%)	10273/10293 (99.8%)	37744/37896 (99.6%)	1177/1177 (100.0%)	3163/3207 (98.6%)	520/520 (100%)
EOSINOPHILS (10^9 l^{-1})	22452/22857 (98.2%)	10272/10293 (99.8%)	37736/37896 (99.6%)	1177/1177 (100.0%)	3163/3207 (98.6%)	520/520 (100%)
BASOPHILS (10^9 l^{-1})	22448/22857 (98.2%)	10270/10293 (99.8%)	37745/37896 (99.6%)	1177/1177 (100.0%)	3162/3207 (98.6%)	520/520 (100%)
SODIUM (mM)	22442/22857 (98.2%)	9664/10293 (93.9%)	36409/37896 (96.1%)	1173/1177 (99.7%)	3180/3207 (99.2%)	
ALBUMIN (g/L)	20010/22857 (87.5%)	8783/10293 (85.3%)	35625/37896 (94.0%)	1160/1177 (98.6%)	3027/3207 (94.4%)	
ALKALINE PHOSPHATASE (IU/L)	19885/22857 (87.0%)	8799/10293 (85.5%)	35604/37896 (94.0%)	1111/1177 (94.4%)	3017/3207 (94.1%)	
ALT (IU/L)	19692/22857 (86.2%)	8689/10293 (84.4%)	35547/37896 (93.8%)	1037/1177 (88.1%)	3003/3207 (93.6%)	

UREA (mM)	22400/22857 (98.0%)	9667/10293 (93.9%)	36398/37896 (96.0%)	1141/1177 (96.9%)	3176/3207 (99.0%)	
BILIRUBIN (umol/L)	19705/22857 (86.2%)	8716/10293 (84.7%)	35550/37896 (93.8%)	940/1177 (79.9%)	3006/3207 (93.7%)	
CREATININE (umol/L)	22457/22857 (98.2%)	9655/10293 (93.8%)	36415/37896 (96.1%)	1172/1177 (99.6%)	3181/3207 (99.2%)	
eGFR (ml/min)	22405/22857 (98.0%)	9649/10293 (93.7%)	36415/37896 (96.1%)	1172/1177 (99.6%)	3171/3207 (98.9%)	
POTASSIUM (mM)	22043/22857 (96.4%)	9306/10293 (90.4%)	34910/37896 (92.1%)	1057/1177 (89.8%)	3105/3207 (96.8%)	
CRP (mg/L)	19068/22857 (83.4%)	8204/10293 (79.7%)	35245/37896 (93.0%)	1136/1177 (96.5%)	2829/3207 (88.2%)	
Respiratory Rate (breath/min)	22794/22857 (99.7%)	1177/1177 (100.0%)	10091/10293 (98.0%)	33459/37896 (88.3%)	3204/3207 (99.9%)	520/520 (100%)
Heart Rate (beats/min)	22845/22857 (99.9%)	1176/1177 (99.9%)	10117/10293 (98.3%)	33461/37896 (88.3%)	3206/3207 (100.0%)	520/520 (100%)
Systolic Blood Pressure (mmHg)	22843/22857 (99.9%)	1171/1177 (99.5%)	10083/10293 (98.0%)	33459/37896 (88.3%)	3207/3207 (100.0%)	520/520 (100%)
Diastolic Blood Pressure (mmHg)	22841/22857 (99.9%)	1171/1177 (99.5%)	10082/10293 (98.0%)	33459/37896 (88.3%)	3207/3207 (100.0%)	520/520 (100%)
Oxygen Saturation (%)	22837/22857 (99.9%)	1177/1177 (100.0%)	10118/10293 (98.3%)	33459/37896 (88.3%)	3207/3207 (100.0%)	520/520 (100%)
Tympanic Temperature (C)	22767/22857 (99.6%)	1177/1177 (100.0%)	10115/10293 (98.3%)	33456/37896 (88.3%)	3207/3207 (100.0%)	520/520 (100%)

Appendix D:
Prospective & External Evaluation of CURIAL-Rapide & CURIAL-Lab

Supplementary Table S6: Evaluation of the performance of (a) CURIAL-Rapide and (b) CURIAL-Lab, calibrated during training to achieve sensitivities of 80% and 90%, on an independent prospective set of all admissions to OUH during the second-wave of COVID-19, between October 1, 2020 and March 6, 2021. (c) Benchmark performance of CURIAL-1.0 (Soltan et al. 2020) on the prospective set. Mean values are reported alongside SD across the three imputation methods.

Model	(a) CURIAL-Rapide: FBC + Vitals		(b) CURIAL-Lab: FBC, U&E, LF Tests, CRP + Vitals		(c) CURIAL-1.0: Blood Tests + Blood Gas + Vitals	
Calibration	80%	90%	80%	90%	80%	90%
Sensitivity	74.7 (0.4)	85.6 (0.6)	72.9 (0.8)	85.7 (0.9)	73.6 (0.3)	85.9 (1.4)
Specificity	78.6 (0.4)	59.1 (0.3)	87.3 (1.1)	68.6 (2.2)	86.6 (0.4)	67.1 (0.8)
PPV	14.9 (0.1)	9.46 (0.0)	22.4 (1.2)	12.0 (0.6)	21.5 (0.4)	11.6 (0.0)
NPV	98.4 (0.0)	98.8 (0.0)	98.5 (0.0)	99.0 (0.0)	98.5 (0.0)	99.0 (0.1)
F1	0.248 (0.003)	0.170 (0.001)	0.342 (0.014)	0.211 (0.009)	0.331 (0.005)	0.204 (0.001)
AUROC	0.843 (0.002)	0.843 (0.002)	0.878 (0.001)	0.878 (0.001)	0.875 (0.002)	0.875 (0.002)

External validation at independent NHS Trusts:

We externally validated CURIAL-Rapide and CURIAL-Lab by applying the respective models to results of first-available blood test results and vital signs (Figure 1B), comparing model predictions to confirmatory SARS-CoV-2 viral genome test results. For trusts where blood-gas results were available for electronic extraction, we also evaluated CURIAL-1.0. Patients meeting inclusion criteria had an unscheduled acute or emergency care admission, during the specified periods, received a blood draw on arrival and were aged over 18. We excluded patients who did not have a valid

confirmatory test result within a prespecified period, or who had opted out of EHR research. Screening against eligibility criteria, followed by anonymisation, was performed by the respective NHS Trusts.

Evaluation at Portsmouth Hospitals University NHS Trust (PUH) considered all patients admitted to the Queen Alexandria Hospital, serving a population of 675,000 and offering tertiary referral services to the surrounding region, between March 1, 2020 and February 28, 2021. Confirmatory COVID-19 testing was by laboratory SARS-CoV-2 RT-PCR assay (Ct for positive result ≤36), considering any positive PCR result within 48hrs of admission as a true positive. As blood gas results were not available for electronic extraction, we evaluated only CURIAL-Rapide and CURIAL-Lab at PUH.

Evaluation at University Hospitals Birmingham NHS Foundation (UHB) trust considered all patients admitted to The Queen Elizabeth Hospital, Birmingham, between December 01, 2019 and October 29, 2020. The Queen Elizabeth Hospital is a large tertiary referral unit within the UHB group which provides healthcare services for a population of 2.2 million across the West Midlands. Confirmatory COVID-19 testing was performed by laboratory SARS-CoV-2 RT-PCR assay (Ct for positive result ≤36).

Evaluation at Bedfordshire Hospitals NHS Foundation Trust (BHT) considered all patients admitted to Bedford Hospital between January 1, 2021 and March 31, 2021. BHT provides healthcare services for a population of around 620,000 in Bedfordshire. Confirmatory COVID-19 testing was performed on the day of admission by point-of-care PCR based nucleic acid testing [SAMBA-II & Panther Fusion System, Diagnostics in the Real World, UK, and Hologic, USA]. The Ct for a positive clinical result was ≤36. In an evaluation of the SAMBA-II against laboratory RT-PCR testing, the SAMBA-II achieved sensitivity of 96.9% and specificity of 99.1%.[2,3]

We report sensitivity, specificity, positive and negative predictive values (PPV and NPV), AUROC and F1 alongside 95% CIs (Supplementary Table S7 & Figure 2), comparing model predictions to results of confirmatory viral testing (laboratory PCR and SAMBA-II). 95% Confidence intervals for sensitivity, specificity and predictive values were computed using Wilson's Method,[4] and for AUROC with DeLong's method.[5]

Supplementary Table S7: Performance of CURIAL-Rapide, CURIAL-Lab & CURIAL-1.0 (Soltan et al. 2020) during external validation at three UK Hospital trusts. All models were calibrated during training to achieve 90% sensitivity. Results are reported alongside 95% confidence intervals. (Acronyms – FBC: Complete Blood Count, U&E: Creatinine & Electrolytes, LFD: Liver Function Test, CRP: C-Reactive Protein)

	**Portsmouth Hospitals University NHS Trust** ***n= 37,896, prevalence = 5.29%***		**University Hospitals Birmingham NHS Foundation Trust** ***n=10,293; prevalence = 4.27%***			**Bedfordshire Hospitals NHS Foundation Trust** ***n=1,177; prevalence = 12.2%***		
	CURIAL-Rapide	CURIAL-Lab	CURIAL-Rapide	CURIAL-Lab	CURIAL-1.0	CURIAL-Rapide	CURIAL-Lab	CURIAL-1.0
Sensitivity (%)	83.5 (81.8 - 85.1)	84.1 (82.5 - 85.7)	82.2 (78.4 - 85.5)	78.8 (74.8 - 82.4)	83.4 (79.6 - 86.6)	74.3 (66.6 - 80.7)	74.3 (66.6 - 80.7)	72.9 (65.1 - 79.5)
Specificity (%)	63.6 (63.1 - 64.1)	71.3 (70.9 - 71.8)	65.4 (64.5 - 66.3)	74.7 (73.8 - 75.5)	68.7 (67.7 - 69.6)	81.8 (79.3 - 84.0)	84.8 (82.5 - 86.9)	83.6 (81.3 - 85.8)

PPV (%)	11.4 (10.9 - 11.9)	14.1 (13.5 - 14.7)	9.6 (8.7 - 10.6)	12.2 (11.0 - 13.4)	10.6 (9.6 - 11.7)	36.3 (31.0 - 41.9)	40.5 (34.8 - 46.5)	38.3 (32.8 - 44.2)
NPV (%)	98.6 (98.4 - 98.7)	98.8 (98.6 - 98.9)	98.8 (98.5 - 99.0)	98.8 (98.5 - 99.0)	98.9 (98.7 - 99.2)	95.8 (94.3 - 96.9)	95.9 (94.5 - 97.0)	95.7 (94.2 - 96.8)
F1	0.200	0.241	0.172	0.211	0.188	0.487	0.525	0.502
AUROC	0.842 (0.832 - 0.852)	0.872 (0.863 - 0.882)	0.836 (0.814 - 0.858)	0.858 (0.838 - 0.878)	0.846 (0.825 - 0.867)	0.854 (0.819 - 0.889)	0.881 (0.851 - 0.912)	0.865 (0.830 - 0.900)

Comparison with Lateral Flow Tests

We considered any positive lateral flow test which was followed by a positive PCR test within a +/- 48hr window of a patient being admitted to hospital to represent a true positive infection. As previously, model predictions were generated using blood tests performed from the first blood draw on arrival and first-recorded vital signs. In the integrated clinical pathway (Figure 1C), patients were considered COVID-19-suspected if they had either a positive LFD result or CURIAL prediction. Results are show in Figure 3 and Supplementary Table S8.

Supplementary Table S8: Performance characteristics of (a) INNOVA SARS-CoV2 Rapid Antigen Tests, (b) CURIAL-Rapide & CURIAL-Lab, calibrated during training to a sensitivity of 80%, and (c) combined clinical pathways considering either a positive CURIAL-Rapide/CURIAL-Lab result or a positive LFD test as a COVID-19 suspected case, at Oxford University Hospitals NHS Foundation Trust between December 23, 2020 & March 6, 2021. Error bars show 95% confidence intervals.

n=3207 prevalence 11.1%		**CURIAL-Rapide: FBC & Vitals**		**CURIAL-Lab: FBC, U&E, LF Tests, CRP + Vitals**		**CURIAL-1.0: Blood Tests + Blood Gas + Vitals**	
Feature Sets	Innova SARS-CoV-2 Rapid Antigen Tests	CURIAL-Rapide	Innova Lateral Flow Tests + CURIAL-Rapide	CURIAL-Lab	Innova Lateral Flow Tests + CURIAL-Lab	CURIAL-1.0	Innova Lateral Flow Tests + CURIAL-1.0
**Sensitivity**	56.9% (51.7 - 62.0)	78.0% (73.4 - 82.0)	88.2% (84.4 - 91.1)	74.4% (69.6 - 78.6)	85.6% (81.6 - 88.9)	76.1% (71.4 - 80.2)	85.9% (81.9 - 89.2)
**Specificity**	99.8% (99.6 - 99.9)	80.0% (78.5 - 81.4)	79.9% (78.4 - 81.3)	88.4% (87.2 - 89.5)	88.3% (87.0 - 89.4)	88.5% (87.3 - 89.6)	88.4% (87.1 - 89.5)
**PPV**	97.6% (94.5 - 99.0)	32.7% (29.6 - 35.9)	35.3% (32.2 - 38.5)	44.4% (40.4 - 48.4)	47.6% (43.7 - 51.4)	45.2% (41.2 - 49.2)	47.9% (44.0 - 51.8)
**NPV**	94.9% (94.1 - 95.6)	96.7% (95.9 - 97.3)	98.2% (97.6 - 98.7)	96.5% (95.7 - 97.2)	98.0% (97.4 - 98.5)	96.7% (96.0 - 97.4)	98.1% (97.4 - 98.5)
**F1**	0.719	0.461	0.504	0.556	0.612	0.567	0.615
**AUROC**		0.854 (0.829 - 0.879)	0.919 (0.899 - 0.940)	0.877 (0.853 - 0.901)	0.925 (0.905 - 0.945)	0.887 (0.865 - 0.909)	0.926 (0.907 - 0.946)

Appendix E:
CURIAL-Rapide lab-free deployment

Deployment of the OLO haematology analyser & CURIAL-Rapide operated between February 18, 2021 and May 10, 2021 between 8am and 8pm.

Operator Training

We specified that clinical staff carrying out the service evaluation must ordinarily be employed by OUH, participate in the care of patients as part of their usual duties, have completed all statutory & mandatory training required by the trust for their role including for electronic health record systems, and be familiar and competent in

using these systems as part of their usual role. We permitted student doctors meeting the above requirements to participate. Training to operate the OLO was provided by in-person device training, supported by demonstration and documentation from the device manufacturers, and a supporting online training video (made available at https://youtu.be/UofBAL7sAzc). Weekly quality-control checks were performed on the OLO analysers.

Enrolment:
OUH sites for eligibility: John Radcliffe Hospital
Inclusion: Adult patients (aged >18)
Clinical areas for sampling eligibility were ED Assessment area, ED Majors Beds and ED Resus. Patients who are not receiving blood tests on presentation to the emergency department as part of their care were not eligible.

Process:
Eligible patients were identified to take part in the service evaluation using the locally-adopted Cerner FirstNet system. Vital signs and blood draws were performed on arrival to the emergency department by healthcare professionals as part of routine care. Following trust procedures, vital signs were documented on the trust electronic health record [SEND; Sensyne Health], and blood bottles were labelled using printed labels from the electronic record. Two drops of venous blood (27uL) from a routinely-collected EDTA blood tube were extracted using a single-use sampling device, and prepared for OLO analysis by trained operators directed by on-screen instructions.[6] OLO results were uploaded immediately to the electronic medical record using the POCcelerator Data Management System [Siemens Healthineers GmbH, Erlangen, Germany], making results available to clinicians and supporting routine patient care. Routine laboratory FBC analysis [Sysmex XN Automated, Sysmex UK] was used to confirm point of care results. Clinical care followed existing pathways and departmental procedures.

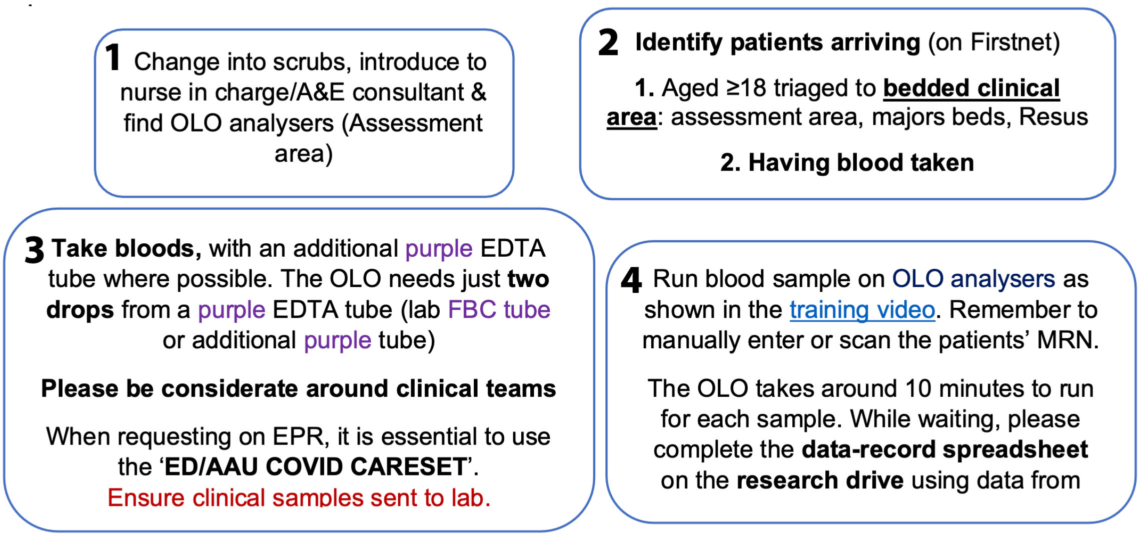

Supplementary Figure S3: Instructions to trained operators, specifying eligibility criteria for the service evaluation, sample handling and processing techniques.

Confirmatory COVID-19 Testing:

Confirmatory testing of patients enrolled in the OLO/CURIAL-Rapide service evaluation, and LFD comparison, followed OUH trust policies. Swabs of the nose and throat were routinely performed in the emergency department for all patients being admitted to OUH. Lateral Flow Testing (Innova SARS-CoV-2 Antigen Rapid Qualitative Test) was performed in the department, by trained nursing or medical staff, and results were documented on the electronic record. Swabs for PCR were transferred to the clinical laboratory in viral transport medium and tested by PCR (ThermoFisher TaqPath). Where patients were not tested for COVID-19 by confirmatory PCR, or did not receive blood tests or vital signs as part of routine care, we excluded the patients from the CURIAL-Rapide evaluation. We also excluded patients with an invalid OLO result and no subsequent successful result, thereby ensuring data completeness.

Analysis

Binary CURIAL-Rapide triage predictions (COVID-19-Suspected and COVID-19-Negative) were generated using a custom Python 3.0 application. Libraries used included scikit-learn, pandas, and NumPy. No other clinical data was made available to the algorithm. CURIAL-Rapide predictions were not made available to physician in this study, so as not to influence the clinical triage category or decisions to proceed to confirmatory testing.

We compared CURIAL-Rapide predictions, lateral flow results, and clinical triage category by first-assessing physician against a PCR reference standard. Assessing physicians were trained to use the Green/Amber/Blue categorization system as part of their ordinary clinical role. We determined and report sensitivity, specificity, PPV, NPV and accuracy, alongside 95% confidence intervals. We calculated the time-to-result for each test, presenting mean with standard deviation for normally distribution data, and median with interquartile range for data with a skewed distributed. Laboratory FBC samples were not processed for 2 of the 520 patients, owing to sample or labelling errors. For paired samples, we compared time-to-result between each test using a one-tailed Wilcoxon Signed Rank test. We additionally performed a Kaplan-Meier survival analysis (Figure 4). Analyses were performed in Python 3.0 using scikit-learn (v0.24) and pandas (v1.3.3).

We report our study in compliance with the “Standards for Reporting Diagnostic accuracy studies” (STARD) standards.[7,8]

Appendix F:

Explainability: Understanding the role of predictors

To understand the role of predictors in model performance we performed relative feature importance analysis, showing the weights of individual features in the trained models. A limitation of this analysis is that where features are highly correlated, any one of the correlated features may be selected during training, ascribed importance and thereafter the relative importance of highly correlated features may appear to be decreased. Therefore, to better understand the impact of individual predictors on model performance, we performed SHAP (SHapley Additive exPlanations) analysis using the prospective OUH second-wave test set. An advantage of this analysis is that SHAP values can be used to interpret the impact on model prediction of the value of a given feature, in comparison to a baseline value.[9]

As previously, the features which were most important to CURIAL-1.0 model predictions were Basophils, Eosinophils, CRP, and Oxygen requirements. SHAP analysis found that similar features were most important to model predictions, with higher relative importance for CRP when compared to Basophils. The highest ranking features, both in relative feature importance analysis and by SHAP scores, were similar between CURIAL-1.0 and CURIAL-Lab. For CURIAL-Rapide, granulocyte counts (Basophils & Eosinophils) were expectedly amongst the highest ranking features, alongside Oxygen saturations and respiratory rate.

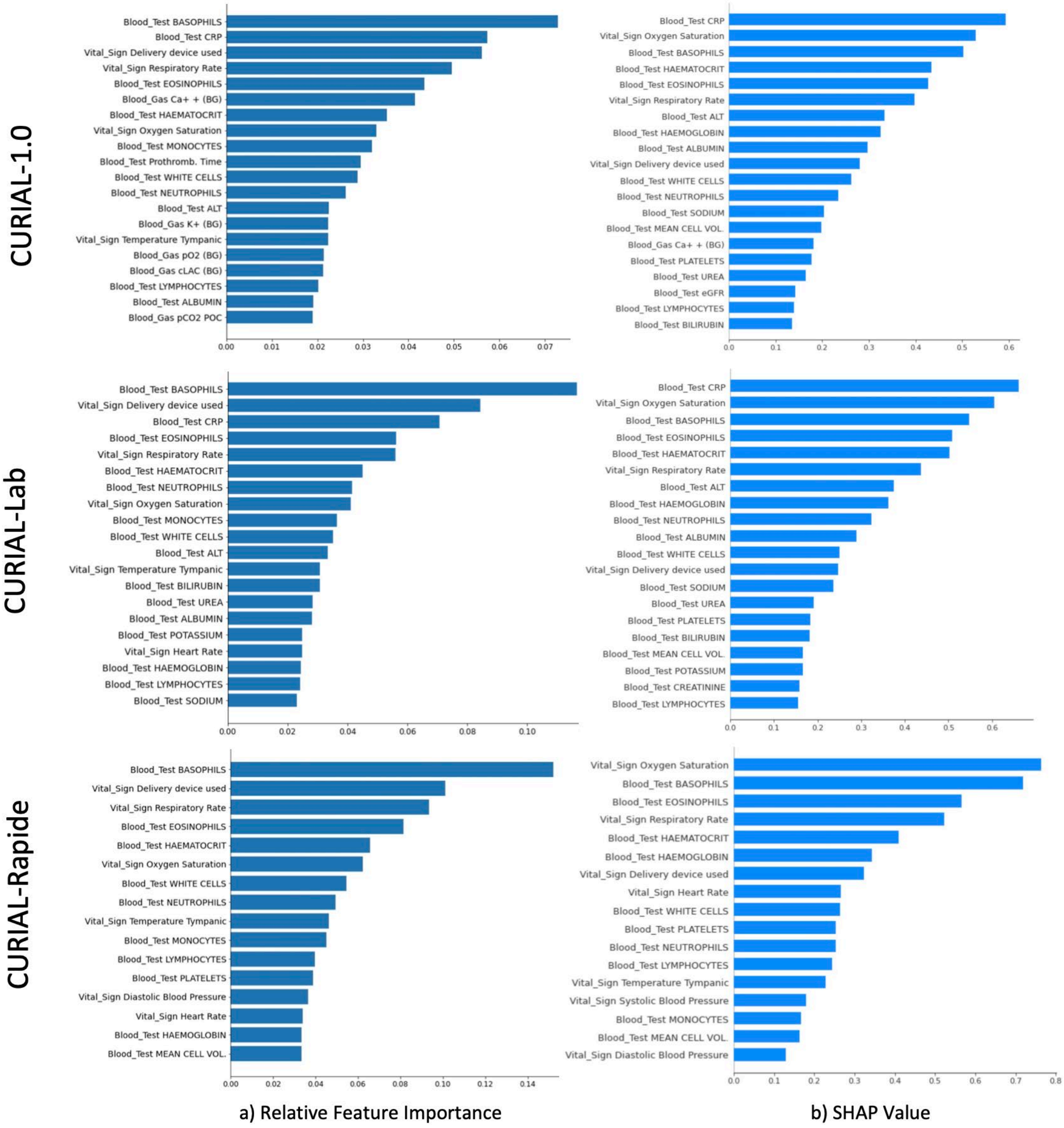

Supplementary Figure S4: Explainability analyses for CURIAL-1.0, CURIAL-Lab & CURIAL-Rapide. a) Relative feature importance of individual predictors within the trained models, b) SHAP (Shapley Additive Explanations) score analysis on the OUH second wave prospective set.

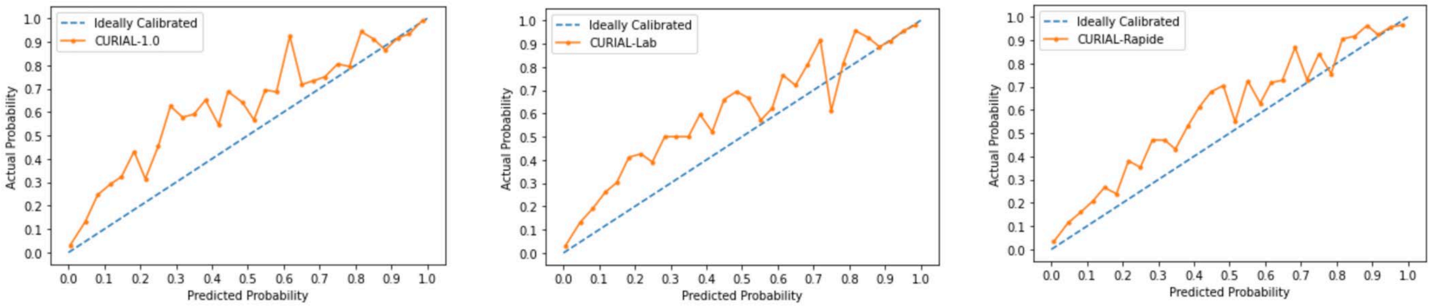

Supplementary Figure S6: Calibration curve analysis demonstrating calibration of CURIAL-1.0, CURIAL-Lab & CURIAL-Rapide during the OUH second wave prospective evaluation.

Appendix G: Supplementary Results

Supplementary Table S9: Subgroup performance by gender for CURIAL-Rapide, CURIAL-Lab & CURIAL-1.0 (Soltan et al.) during external validation at three UK Hospital trusts. Results are reported alongside 95% confidence intervals.

		Portsmouth Hospitals University NHS Trust *n= 37,896, prevalence = 5.29%*		University Hospitals Birmingham NHS Foundation Trust *n=10,293; prevalence = 4.27%*			Bedfordshire Hospitals NHS Foundation Trust *n=1,177; prevalence = 12.2%*		
		CURIAL-Rapide	CURIAL-Lab	CURIAL-Rapide	CURIAL-Lab	CURIAL-1.0	CURIAL-Rapide	CURIAL-Lab	CURIAL-1.0
Male	Sensitivity (%)	85.3% (83.1 - 87.3)	87.2% (85.0 - 89.1)	84.4% (79.5 - 88.4)	81.7% (76.5 - 86.0)	86.8% (82.1 - 90.4)	70.7% (58.0 - 80.8)	79.3% (67.2 - 87.7)	75.9% (63.5 - 85.0)
	Specificity (%)	62.8% (62.1 - 63.6)	68.5% (67.8 - 69.2)	66.1% (64.7 - 67.4)	74.3% (73.0 - 75.6)	67.3% (66.0 - 68.7)	81.4% (78.0 - 84.4)	85.1% (81.9 - 87.8)	83.3% (80.0 - 86.1)
	PPV (%)	13.0% (12.2 - 13.8)	15.2% (14.3 - 16.2)	12.3% (10.8 - 13.9)	15.2% (13.4 - 17.1)	13.0% (11.5 - 14.7)	27.9% (21.3 - 35.6)	35.1% (27.5 - 43.6)	31.7% (24.5 - 39.8)
	NPV (%)	98.5% (98.3 - 98.7)	98.8% (98.6 - 99.0)	98.7% (98.2 - 99.0)	98.6% (98.2 - 99.0)	98.9% (98.5 - 99.2)	97.6% (95.8 - 98.6)	97.6% (95.8 - 98.6)	97.1% (95.2 - 98.3)
	F1	0.225	0.259	0.214	0.256	0.226	0.400	0.487	0.447
	AUROC	0.851 (0.837 - 0.864)	0.881 (0.869 - 0.894)	0.853 (0.826 - 0.88)	0.873 (0.849 - 0.897)	0.865 (0.841 - 0.889)	0.84 (0.782 - 0.898)	0.894 (0.848 - 0.939)	0.867 (0.811 - 0.923)
Female	Sensitivity (%)	81.4% (78.8 - 83.8)	80.7% (78.1 - 83.1)	79.1% (72.6 - 84.4)	74.7% (67.9 - 80.5)	78.6% (72.1 - 83.9)	76.7% (66.8 - 84.4)	70.9% (60.6 - 79.5)	70.9% (60.6 - 79.5)
	Specificity (%)	64.0% (63.3 - 64.7)	73.3% (72.7 - 73.9)	64.8% (63.6 - 66.1)	75.0% (73.8 - 76.2)	69.8% (68.6 - 71.0)	82.3% (78.6 - 85.5)	84.4% (80.9 - 87.5)	84.0% (80.4 - 87.1)
	PPV (%)	9.5% (8.9 - 10.2)	12.3% (11.5 - 13.1)	7.2% (6.1 - 8.4)	9.3% (8.0 - 11.0)	8.2% (7.0 - 9.6)	44.6% (36.8 - 52.6)	45.9% (37.6 - 54.3)	45.2% (37.0 - 53.6)
	NPV (%)	98.7% (98.5 - 98.9)	98.8% (98.6 - 99.0)	98.9% (98.5 - 99.2)	98.9% (98.5 - 99.1)	99.0% (98.6 - 99.2)	95.0% (92.4 - 96.7)	94.0% (91.3 - 95.9)	94.0% (91.2 - 95.9)
	F1	0.170	0.213	0.132	0.166	0.149	0.564	0.557	0.552
	AUROC	0.83 (0.815 - 0.845)	0.859 (0.844 - 0.873)	0.811 (0.774 - 0.848)	0.834 (0.8 - 0.869)	0.817 (0.781 - 0.854)	0.863 (0.819 - 0.907)	0.871 (0.83 - 0.913)	0.863 (0.817 - 0.909)

Supplementary Table S10: Subgroup performance by ethnicity for CURIAL-Rapide, CURIAL-Lab & CURIAL-1.0 (Soltan et al.) during external validation at three UK

Hospital trusts. Subgroups are shown where group size is ≥15 or 0.25% of the evaluation population. Results are reported alongside 95% confidence intervals.

		Portsmouth Hospitals University NHS Trust *n= 37,896, prevalence = 5.29%*		University Hospitals Birmingham NHS Foundation Trust *n=10,293; prevalence = 4.27%*			Bedfordshire Hospitals NHS Foundation Trust *n=1,177; prevalence = 12.2%*		
		CURIAL-Rapide	CURIAL-Lab	CURIAL-Rapide	CURIAL-Lab	CURIAL-1.0	CURIAL-Rapide	CURIAL-Lab	CURIAL-1.0
White	Sensitivity (%)	82.4% (80.3 - 84.2)	82.6% (80.6 - 84.5)	80.7% (75.1 - 85.3)	75.9% (69.9 - 81.0)	81.1% (75.6 - 85.7)	72.8% (64.0 - 80.1)	72.8% (64.0 - 80.1)	71.1% (62.1 - 78.6)
	Specificity (%)	63.7% (63.1 - 64.2)	71.3% (70.8 - 71.8)	62.7% (61.5 - 63.9)	73.0% (71.9 - 74.0)	66.3% (65.2 - 67.4)	81.4% (78.8 - 83.8)	84.9% (82.5 - 87.1)	84.3% (81.8 - 86.5)
	PPV (%)	10.5% (10.0 - 11.1)	13.0% (12.3 - 13.7)	6.9% (6.0 - 8.0)	8.8% (7.6 - 10.1)	7.7% (6.7 - 8.8)	32.9% (27.4 - 39.0)	37.7% (31.6 - 44.3)	36.2% (30.2 - 42.6)
	NPV (%)	98.6% (98.4 - 98.7)	98.8% (98.6 - 98.9)	99.0% (98.6 - 99.2)	98.9% (98.5 - 99.1)	99.0% (98.7 - 99.3)	96.0% (94.4 - 97.2)	96.1% (94.6 - 97.3)	95.9% (94.3 - 97.0)
	F1	0.187	0.225	0.128	0.158	0.140	0.454	0.497	0.479
	AUROC	0.831 (0.819 - 0.843)	0.865 (0.854 - 0.876)	0.825 (0.794 - 0.856)	0.842 (0.814 - 0.869)	0.822 (0.791 - 0.852)	0.848 (0.809 - 0.887)	0.877 (0.842 - 0.911)	0.857 (0.817 - 0.898)
South Asian	Sensitivity (%)	90.0% (69.9 - 97.2)	80.0% (58.4 - 91.9)	79.2% (70.0 - 86.1)	78.1% (68.9 - 85.2)	81.2% (72.3 - 87.8)	87.5% (64.0 - 96.5)	87.5% (64.0 - 96.5)	87.5% (64.0 - 96.5)
	Specificity (%)	71.4% (63.8 - 78.0)	76.6% (69.3 - 82.6)	72.2% (69.6 - 74.6)	78.5% (76.2 - 80.7)	74.2% (71.7 - 76.6)	81.8% (69.7 - 89.8)	74.5% (61.7 - 84.2)	78.2% (65.6 - 87.1)
	PPV (%)	29.0% (19.2 - 41.3)	30.8% (19.9 - 44.3)	17.8% (14.5 - 21.7)	21.7% (17.7 - 26.3)	19.4% (15.8 - 23.5)	58.3% (38.8 - 75.5)	50.0% (32.6 - 67.4)	53.8% (35.5 - 71.2)
	NPV (%)	98.2% (93.7 - 99.5)	96.7% (91.9 - 98.7)	97.8% (96.7 - 98.6)	97.9% (96.8 - 98.6)	98.1% (97.0 - 98.8)	95.7% (85.8 - 98.8)	95.3% (84.5 - 98.7)	95.6% (85.2 - 98.8)
	F1	0.439	0.444	0.291	0.339	0.313	0.700	0.636	0.667
	AUROC	0.919 (0.855 - 0.984)	0.896 (0.806 - 0.986)	0.824 (0.771 - 0.877)	0.855 (0.808 - 0.902)	0.856 (0.81 - 0.902)	0.888 (0.792 - 0.983)	0.9 (0.825 - 0.975)	0.926 (0.857 - 0.995)
Black	Sensitivity (%)	90.9% (62.3 - 98.4)	90.9% (62.3 - 98.4)	90.5% (71.1 - 97.3)	85.7% (65.4 - 95.0)	90.5% (71.1 - 97.3)	62.5% (30.6 - 86.3)	62.5% (30.6 - 86.3)	62.5% (30.6 - 86.3)
	Specificity (%)	59.4% (52.1 - 66.3)	64.4% (57.2 - 71.1)	67.2% (62.8 - 71.3)	74.5% (70.4 - 78.3)	71.3% (67.0 - 75.2)	85.7% (68.5 - 94.3)	96.4% (82.3 - 99.4)	82.1% (64.4 - 92.1)
	PPV (%)	12.0% (6.7 - 20.8)	13.5% (7.5 - 23.1)	11.1% (7.2 - 16.7)	13.2% (8.5 - 20.0)	12.5% (8.2 - 18.7)	55.6% (26.7 - 81.1)	83.3% (43.6 - 97.0)	50.0% (23.7 - 76.3)
	NPV (%)	99.1% (94.9 - 99.8)	99.1% (95.3 - 99.8)	99.4% (97.7 - 99.8)	99.1% (97.5 - 99.7)	99.4% (97.8 - 99.8)	88.9% (71.9 - 96.1)	90.0% (74.4 - 96.5)	88.5% (71.0 - 96.0)
	F1	0.213	0.235	0.198	0.229	0.220	0.588	0.714	0.556
	AUROC	0.908 (0.813 - 1.000)	0.899 (0.795 - 1.000)	0.844 (0.775 - 0.912)	0.899 (0.84 - 0.959)	0.872 (0.788 - 0.956)	0.763 (0.537 - 0.99)	0.857 (0.665 - 1.000)	0.79 (0.573 - 1.000)
Chinese	Sensitivity (%)			100.0% (34.2 - 100.0)	50.0% (9.5 - 90.5)	100.0% (34.2 - 100.0)			
	Specificity (%)			76.9% (61.7 - 87.4)	74.4% (58.9 - 85.4)	71.8% (56.2 - 83.5)			
	PPV (%)			18.2% (5.1 - 47.7)	9.1% (1.6 - 37.7)	15.4% (4.3 - 42.2)			
	NPV (%)			100.0% (88.6 - 100.0)	96.7% (83.3 - 99.4)	100.0% (87.9 - 100.0)			
	F1			0.308	0.154	0.267			
	AUROC			0.91 (0.766 - 1.000)	0.769 (0.31 - 1.000)	0.987 (0.952 - 1.00)			
Other	Sensitivity (%)	81.8% (61.5 - 92.7)	81.8% (61.5 - 92.7)	90.0% (69.9 - 97.2)	95.0% (76.4 - 99.1)	95.0% (76.4 - 99.1)	100.0% (51.0 - 100.0)	100.0% (51.0 - 100.0)	100.0% (51.0 - 100.0)
	Specificity (%)	70.5% (64.6 - 75.8)	77.3% (71.7 - 82.0)	77.3% (72.4 - 81.6)	82.7% (78.2 - 86.5)	82.7% (78.2 - 86.5)	84.0% (65.3 - 93.6)	80.0% (60.9 - 91.1)	72.0% (52.4 - 85.7)

	PPV (%)	19.6% (12.7 - 28.8)	24.0% (15.8 - 34.8)	20.2% (13.2 - 29.7)	26.0% (17.3 - 37.1)	21.3% (14.1 - 31.0)	50.0% (21.5 - 78.5)	44.4% (18.9 - 73.3)	36.4% (15.2 - 64.6)
	NPV (%)	97.8% (94.5 - 99.1)	98.0% (94.9 - 99.2)	99.2% (97.1 - 99.8)	99.6% (97.9 - 99.9)	99.6% (97.7 - 99.9)	100.0% (84.5 - 100.0)	100.0% (83.9 - 100.0)	100.0% (82.4 - 100.0)
	F1	0.316	0.371	0.330	0.409	0.349	0.667	0.615	0.533
	AUROC	0.861 (0.763 - 0.96)	0.915 (0.856 - 0.974)	0.933 (0.87 - 0.996)	0.962 (0.926 - 0.998)	0.954 (0.912 - 0.996)	1.0 (0.890 - 1.000)	0.95 (0.861 - 1.000)	0.96 (0.882 - 1.000)
Mixed	Sensitivity (%)	90.0% (59.6 - 98.2)	90.0% (59.6 - 98.2)	100.0% (43.9 - 100.0)	66.7% (20.8 - 93.9)	100.0% (43.9 - 100.0)			
	Specificity (%)	67.2% (58.7 - 74.7)	74.2% (66.0 - 81.0)	70.5% (63.1 - 76.9)	75.3% (68.2 - 81.2)	73.5% (66.3 - 79.6)			
	PPV (%)	17.6% (9.6 - 30.3)	21.4% (11.7 - 35.9)	5.8% (2.0 - 15.6)	4.7% (1.3 - 15.5)	6.4% (2.2 - 17.2)			
	NPV (%)	98.9% (93.8 - 99.8)	99.0% (94.3 - 99.8)	100.0% (96.8 - 100.0)	99.2% (95.6 - 99.9)	100.0% (96.9 - 100.0)			
	F1	0.295	0.346	0.109	0.0870	0.12			
	AUROC	0.915 (0.825 - 1.000)	0.859 (0.678 - 1.000)	0.96 (0.89 - 1.000)	0.845 (0.587 - 1.000)	0.906 (0.801 - 1.000)			
Not Stated	Sensitivity (%)	86.3% (82.9 - 89.0)	88.5% (85.4 - 91.0)	85.5% (75.3 - 91.9)	84.1% (73.7 - 90.9)	87.0% (77.0 - 93.0)			
	Specificity (%)	62.5% (61.4 - 63.5)	70.4% (69.4 - 71.3)	69.0% (66.0 - 71.8)	78.8% (76.2 - 81.3)	72.4% (69.5 - 75.1)			
	PPV (%)	12.4% (11.3 - 13.5)	15.5% (14.2 - 16.8)	16.1% (12.7 - 20.2)	21.6% (17.1 - 27.0)	18.0% (14.2 - 22.4)			
	NPV (%)	98.7% (98.3 - 98.9)	99.0% (98.7 - 99.2)	98.6% (97.4 - 99.2)	98.6% (97.5 - 99.2)	98.8% (97.7 - 99.3)			
	F1	0.216	0.263	0.271	0.344	0.298			
	AUROC	0.865 (0.846 - 0.884)	0.887 (0.87 - 0.905)	0.874 (0.823 - 0.924)	0.888 (0.841 - 0.935)	0.889 (0.843 - 0.934)			

Supplementary Table S11: Performance of CURIAL-Rapide, CURIAL-Lab & CURIAL-1.0 (Soltan et al.) on severity subgroups of patients a) admitted to ICU, b) not requiring ICU level care, and c) who were discharged. Results are reported alongside 95% confidence intervals.

		**Oxford University Hospitals** – Prospective Evaluation		
		CURIAL-Rapide	CURIAL-Lab	CURIAL-1.0
Admitted to ICU	Sensitivity (%)	94.6% (89.3 - 97.4)	94.6% (89.3 - 97.4)	96.2% (91.3 - 98.3)
	Specificity (%)	62.7% (59.1 - 66.1)	71.6% (68.2 - 74.7)	67.4% (63.9 - 70.7)
	PPV (%)	30.8% (26.4 - 35.4)	36.8% (31.8 - 42.1)	34.1% (29.4 - 39.1)
	NPV (%)	98.5% (97.0 - 99.3)	98.7% (97.3 - 99.4)	99.0% (97.7 - 99.6)
	F1	0.464	0.530	0.503
	AUROC	0.936 (0.91 - 0.961)	0.955 (0.93 - 0.979)	0.958 (0.933 - 0.983)
Admitted to non-ICU Clinical Area	Sensitivity (%)	83.3% (81.5 - 85.0)	83.3% (81.5 - 84.9)	85.1% (83.4 - 86.7)
	Specificity (%)	63.3% (62.6 - 64.0)	72.6% (72.0 - 73.2)	66.9% (66.2 - 67.5)
	PPV (%)	17.0% (16.2 - 17.8)	21.5% (20.6 - 22.5)	18.8% (18.0 - 19.7)
	NPV (%)	97.7% (97.4 - 97.9)	98.0% (97.7 - 98.2)	98.0% (97.8 - 98.3)
	F1	0.282	0.342	0.308

	AUROC	0.847 (0.836 - 0.858)	0.877 (0.867 - 0.887)	0.873 (0.863 - 0.884)
Discharged	Sensitivity (%)	85.6% (83.2 - 87.7)	84.6% (82.1 - 86.8)	87.1% (84.8 - 89.1)
	Specificity (%)	66.6% (65.6 - 67.6)	75.5% (74.5 - 76.4)	69.9% (68.9 - 70.9)
	PPV (%)	22.0% (20.7 - 23.4)	27.6% (26.0 - 29.2)	24.2% (22.8 - 25.7)
	NPV (%)	97.7% (97.2 - 98.0)	97.8% (97.4 - 98.1)	98.0% (97.6 - 98.3)
	F1	0.350	0.416	0.379
	AUROC	0.874 (0.860 - 0.888)	0.897 (0.884 - 0.91)	0.895 (0.882 - 0.909)

References

1 Chen T, Guestrin C. XGBoost: a scalable tree boosting system. In: KDD '16: proceedings of the 22nd ACM SIGKDD International Conference on Knowledge Discovery and Data Mining. New York, NY: Association for Computing Machinery, 2016: 785–94.

2 Assennato SM, Ritchie AV, Nadala C, et al. Performance evaluation of the SAMBA II SARS-CoV-2 test for point-of-care detection of SARS-CoV-2. *J Clin Microbiol* 2020; **59:** e01262–20.

3 Collier DA, Assennato SM, Warne B, et al. Point of care nucleic acid testing for SARS-CoV-2 in hospitalized patients: a clinical validation trial and implementation study. *Cell Rep Med* 2020; **1:** 100062.

4 Newcombe RG, Altman DG. Proportions and their differences. In: Altman D, Machin D, Bryant T, Gardner M, eds. Statistics with confidence: confidence intervals and statistical guidelines, 2nd edn. Hoboken, NJ: Wiley, 2000.

5 Sun X, Xu W. Fast implementation of DeLong's algorithm for comparing the areas under correlated receiver operating characteristic curves. *IEEE Signal Process Lett* 2014; **21:** 1389–93.

6 Bachar N, Benbassat D, Brailovsky D, et al. An artificial intelligence-assisted diagnostic platform for rapid near-patient hematology. *Am J Hematol* 2021; **96:** 1264–74.

7 Bossuyt PM, Reitsma JB, Bruns DE, et al. STARD 2015: an updated list of essential items for reporting diagnostic accuracy studies. *BMJ* 2015; **351:** h5527.

8 Liu X, Cruz Rivera S, Moher D, et al. Reporting guidelines for clinical trial reports for interventions involving artificial intelligence: the CONSORT-AI extension. *Nat Med* 2020; **26:** 1364–74.

9 Lundberg SM, Lee S-I. A unified approach to interpreting model predictions. In: Jordan MI, LeCun Y, Solla SA, eds. Advances in neural information processing systems. Red Hook, NY: Curran Associates, 2017.
